# Computational Study on Potentially Active Antibacterial Compounds in Secondary Metabolites of Extremophilic Microorganisms

**DOI:** 10.1002/open.202500460

**Published:** 2025-11-28

**Authors:** Dilong Li, Yanni Wang, Yinhuan Huang, Hui Zhou, Xiaoyun Xia, Wei Huang, Chaojie Wang

**Affiliations:** ^1^ Pharmacy Department Ruian Hospital of Traditional Chinese Medicine Wenzhou China; ^2^ Pharmacy Department The Third Affiliated Hospital of Wenzhou Medical University Wenzhou China; ^3^ Department of Public Health Ruian Hospital of Traditional Chinese Medicine Wenzhou China; ^4^ School of Pharmaceutical Sciences Wenzhou Medical University Wenzhou China

**Keywords:** 16‐membered lactone ring, DFT calculation, extreme microbial secondary metabolites, molecular docking, pharmacokinetic parameters

## Abstract

The density functional theory (DFT) method *ω*B97XD/6‐311+G(2d, p) was employed to perform systematic theoretical calculations and comparative analyses on the geometric structures, spectroscopic properties, frontier molecular orbitals, and molecular electrostatic potentials of potential antibacterial compounds derived from 16‐membered lactone ring‐containing secondary metabolites of extremophiles, as well as midecamycin. The reactivity indices of these compounds were further investigated within the framework of conceptual DFT. Additionally, drug‐likeness was evaluated using two independent pharmacokinetic prediction platforms, and molecular docking simulations were conducted to assess their binding affinities. The results indicate that the carboxyl hydrogen, hydroxyl hydrogen, and carbonyl oxygen atoms in these molecules exhibit relatively high reactivity. Compound **3** displays relatively high chemical reactivity, whereas compounds **6** and **9** demonstrate superior chemical stability combined with significant reactivity. Pharmacokinetic predictions reveal poor Caco‐2 permeability for compounds **8** and **9**, low therapeutic indices for compounds **2** and **3**, and the highest metabolic stability in human liver microsomes for compound **7**. Overall, compound **1** exhibits the highest structural and physicochemical similarity to midecamycin. Compound **1** was evaluated for molecular docking with the 50S ribosomal subunit from *Streptomyces* bacteria; the molecular docking results confirm its distinct binding affinity, despite a slightly higher binding energy. The molecular dynamics simulation results indicate that complex **1** exhibits a Gibbs free energy of −30.76 kJ/mol, further supporting its structural stability.

## Graphical abstract

This study employed density functional theory (DFT) and conceptual DFT (CDFT) to calculate the structures and properties of potential antibacterial compounds derived from 16‐membered lactone ring‐containing secondary metabolites of extremophiles, as well as midecamycin. The calculated properties included infrared (IR), UV–Vis, electronic circular dichroism (ECD), nuclear magnetic resonance (NMR), electrostatic potential, and frontier molecular orbitals. Furthermore, ADME/Tox profiles were predicted to evaluate their pharmacokinetic and safety potential. Molecular docking of the most promising compound **1** confirmed its distinct binding affinity with the 50S ribosomal subunit from *Streptomyces* bacteria. Finally, molecular dynamics simulations demonstrated that the complex **1** exhibits structural stability.

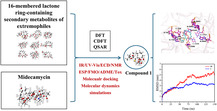

## 1 Introduction

The misuse and overuse of pharmaceuticals, along with environmental drug pollution, have contributed to the emergence of drug resistance in pathogenic microorganisms, particularly bacteria. In light of the growing global threat of antibiotic resistance, the discovery of novel antibacterial compounds has become a critical objective in pharmaceutical research and development. Extreme microorganisms, due to their adaptation to unique ecological environments and extreme survival conditions, possess distinctive biochemical characteristics that enable them to produce structurally novel and functionally diverse secondary metabolites [1]. These metabolites exhibit considerable potential as antibacterial agents. Research has demonstrated that such compounds are effective against infections caused by drug‐resistant pathogens and exhibit low toxicity and minimal side effects, making them promising alternatives to conventional antibacterial agents [2, 3]. Several studies have reported the isolation and identification of antibacterial compounds from the secondary metabolites of extremophilic microorganisms, accompanied by systematic structural and activity analyses [4, 5, 6, 7, 8]. Despite the promising potential of these metabolites, their development into therapeutic agents faces numerous challenges, including the influence of physicochemical properties on biological activity and metabolic stability. Density functional theory (DFT), however, has proven to be a highly efficient and accurate computational tool for predicting molecular properties and binding interactions, thereby significantly reducing experimental time and development costs and facilitating more systematic and rational approach to drug design [9, 10, 11, 12, 13, 14, 15].

Macrolide compounds represent a crucial class of antibacterial agents with widespread clinical applications; however, theoretical investigations of this class remain limited. The Murakami group [16] employed nuclear magnetic resonance (NMR) spectroscopy in conjunction with DFT calculations to reveal significant conformational differences between exiguolide—a marine natural product exhibiting potent anticancer activity—and its parent compound. Hernandes et al. [17] applied DFT to investigate the predominant conformations of azithromycin in aqueous and DMSO environments, providing valuable insights into the molecular interactions between azithromycin and its biological targets. Using DFT analysis, Daher et al. [18] identified key hydrogen bonding interactions that influence the pharmacological activity of solithromycin analogs. Furthermore, Zeng et al. [19] conducted a detailed computational study on the borate affinity mechanism mediated by charged hydrogen bonds between tylosin and 4‐vinylphenylboronic acid (VPBA), employing DFT to elucidate the underlying molecular interactions. Midecamycin is a 16‐membered macrolide antibiotic that has been largely replaced in recent years by newer‐generation macrolides such as azithromycin and clarithromycin [20] due to its cross‐resistance with erythromycin and other antibiotics, as well as its relatively narrow antimicrobial spectrum [21]. However, it has recently regained attention from the scientific community. Research conducted by Wang et al. [22]. demonstrated that acetyl midecamycin exhibited potent in vitro antibacterial activity against all tested strains of Mycoplasma pneumoniae, *Ureaplasma* species, and Mycoplasma hominis strains, with no significant resistance development observed. Further studies [23, 24] have supported the potential of midecamycin or its acetylated derivatives as alternative therapeutic agents for treating M. pneumoniae infections resistant to azithromycin. Additionally, Liu et al. [25] employed liquid chromatography coupled with an electrospray ionization detector to perform quantitative analysis of multiple components in josamycin and midecamycin, thereby providing technical support for the quality control of these related pharmaceuticals.

**FIGURE 1:**
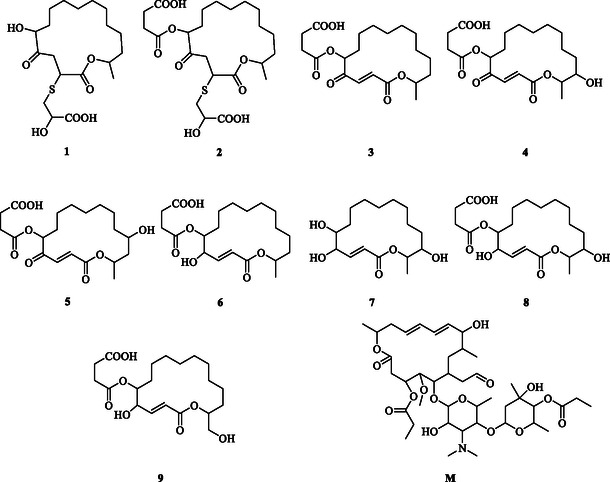
Molecular structures of potential antibacterial compounds derived from 16‐membered lactone ring‐containing secondary metabolites of extremophilic microorganisms, in comparison with midecamycin.

This study investigates the application of DFT in the development of potential antibacterial agents derived from extremophilic microbial secondary metabolites. It systematically examines the relationships among physicochemical properties, structure–activity relationships, and pharmacokinetic (PK) profiles of these compounds, as well as their biological relevance, to provide a theoretical foundation for the discovery of novel antibacterial drugs and to identify promising candidate compounds to address the growing global challenge of antibiotic resistance. The research focuses on nine 16‐membered lactone ring compounds with reported antibacterial activity, as well as midecamycin, as described by the Stierle group [4]. Using DFT, the geometric structures and spectroscopic properties of these compounds were calculated and comparatively analyzed. Potential reactive sites were predicted using conceptual DFT methods. To further investigate the possible binding mode underlying its antibacterial activity, this study performed molecular docking and molecular dynamics (MD) simulations of compound **1** [4], the most active derivative, and compound M with the 50S ribosomal subunit from *Streptomyces* bacteria [26, 27]. Furthermore, the absorption, distribution, metabolism, excretion, and toxicity (ADMET) properties were evaluated using a PK prediction platform to assess drug‐likeness and PK behavior. Compounds with favorable predicted profiles underwent molecular docking against the same receptor as midecamycin, thereby providing theoretical insights into the molecular characteristics of this class of antibacterial agents. The two‐dimensional chemical structures of the selected compounds, including midecamycin (**M,** C_41_H_67_NO_15_, Leucomycin V, 3,4B‐dipropanoate (9 CI, ACI), CAS No. 35 457‐80−8), are presented in Figure 1.

## 2 Computational Methods

In this work, molecular mechanics calculations of compound conformations were performed using the MMFF94 force field, implemented within the GMMX module of GaussView 6.0. Subsequently, DFT‐based calculations were carried out employing the *ω*B97XD [28] functional at the 6‐311+G(2d, p) basis set level to optimize the geometric structures of the nine 16‐membered lactone ring compounds with potential antibacterial activity, as reported by Stierle et al. [4], along with midecamycin, under vacuum conditions. This optimization process yielded stable molecular configurations. Based on the optimized structures, infrared (IR) spectral data were scaled using a correction factor of 0.955 [29, 30]. Ultraviolet–visible absorption spectra (UV–Vis) and electronic circular dichroism spectra (ECD) were computed using the time‐dependent DFT (TD‐DFT) [31, 32]. NMR chemical shifts were calculated using the gauge‐independent atomic orbital (GIAO) method. To account for solvent effects, the PCM [33], as part of the self‐consistent reaction field (SCRF) framework, was applied to simulate aqueous (water, Wat) and methanolic (methanol, Met) environments. Previous investigations have demonstrated that the *ω*B97XD functional yields highly stable geometries [34] and provides spectral predictions that correlate well with experimental observations [35, 36].

The wave function derived from the gas‐phase stable structure was employed in the framework of Conceptual Density Functional Theory (CDFT) to analyze reactivity indices, including the prediction of molecular nucleophilicity and electrophilicity. Global reactivity descriptors—such as ionization potential (IP), electron affinity (EA), electronegativity (*χ*), global hardness (*η*), chemical potential (*μ*), global electrophilicity index (*ω*), and global softness (S)—were computed using the standard definition formulas reported in the literature [37, 38, 39]. Within the CDFT framework, Fukui functions were incorporated to assess the variation in electron density under changes in total electron count or external chemical potential, thereby identifying the reactivity profiles of individual atomic sites [39, 40, 41]. PK properties and toxicological data were predicted using ACD/Labs Percepta software ADMETLab 3.0 platform (https://admetmesh.scbdd.com/) and the ProTox 3.0 (https://tox.charite.de/protox3/). All quantum chemical calculations and analyses were performed with Gaussian 16 [42], Multiwfn 3.8 [43], and VMD software [44]. Molecular docking simulations were performed using AutoDock, and the resulting binding modes were visualized using PyMOL and LigPlot. Additionally, the PrankWeb platform (https://prankweb.cz/) was employed to achieve rapid and accurate prediction of protein ligand‐binding sites [45]. The complexes of compounds M and 1 were subjected to 150 ns MD simulations using GROMACS 2025.3 [46, 47]. The RNA molecule was parameterized with the CHARMM36 force field [48], while the small‐molecule ligands were processed using CGenFF. The RNA–ligand complexes were placed in a cubic water box with periodic boundary conditions; the remaining space was solvated with the TIP3P water model. Electrostatic interactions were treated using the particle mesh Ewald (PME) method, and both van der Waals and Coulomb interactions were calculated with a cutoff distance of 1.0 nm. The systems were subsequently equilibrated in the canonical ensemble (NVT, 310 K) and isothermal–isobaric ensemble (NPT, 1 atm). All bonds involving hydrogen atoms were constrained using the LINCS algorithm to enhance numerical stability and avoid integration instability. Finally, 150 ns production simulations were performed under NPT conditions with a 2 fs integration time step. Binding free energies were computed using the gmx_MMPBSA tool.

## 3 Results and Discussion

### 3.1 Conformational Analysis

**FIGURE 2:**
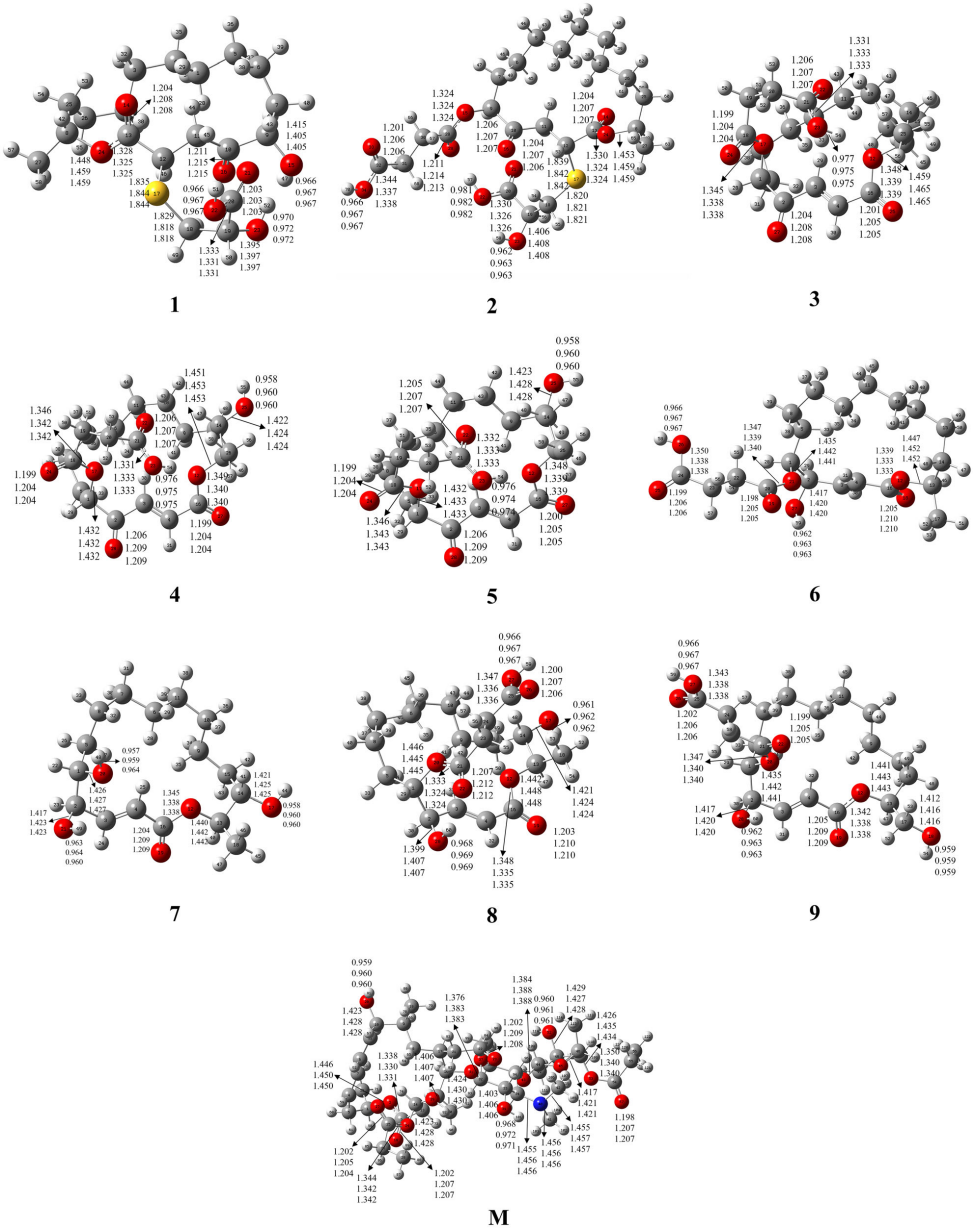
Optimized central bond length values (in Å) of 16‐membered lactone ring compounds exhibiting potential antibacterial activity, derived from secondary metabolites of extremophilic microorganisms, and of midecamycin, calculated using the *ω*B97XD/6‐311+G(2d, p) method under vacuum, aqueous, and methanolic conditions.

As illustrated in Figure 2, conformational search analysis based on the MMFF94 force field reveals that, within the same energy range 3.5 kcal/mol, compounds **1** (C_19_H_32_O_7_S, propanoic acid, 2‐hydroxy‐3‐[[(3R, 6S,16R)‐6‐hydroxy‐16‐methyl‐2,5‐dioxooxacyclohexadec‐3‐yl]thio]‐, (2S)‐ (ACI), CAS No. 2 095 114‐69−3) possess eight rotatable bonds and generates 33 conformers. Compound **2** (C_23_H_36_O_10_S, butanedioic acid, 1‐[(3R, 6S,16R)‐3‐[[(2S)‐2‐carboxy‐2‐hydroxyethyl]thio]‐16‐methyl‐2,5‐dioxooxacyclohexadec‐6‐yl] ester (ACI), CAS No. 2 095 114‐70−6) exhibits 13 rotatable bonds and generates only two distinct conformers. While compound **3** (C_20_H_30_O_7_, butanedioic acid, 1‐[(3E, 6S,16R)‐16‐methyl‐2,5‐dioxooxacyclohexadec‐3‐en‐6‐yl] ester(ACI), CAS No. 56 448‐20−5) has seven, makes 43 conformers. **4** (C_20_H_30_O_8_, butanedioic acid, 1‐[(3E, 6S,15S, 16R)‐15‐hydroxy‐16‐methyl‐2,5‐dioxooxacyclohexadec‐3‐en‐6‐yl] ester(ACI), CAS No. 2 095 114‐71−7), **5** (C_20_H_30_O_8_, butanedioic acid, 1‐[(3E, 6S,16R)‐14‐hydroxy‐16‐methyl‐2,5‐dioxooxacyclohexadec‐3‐en‐6‐yl] ester(ACI), CAS No. 2 095 114‐77−3), and **6** (C_20_H_32_O_7_, butanedioic acid, mono(5‐hydroxy‐16‐methyl‐2‐oxooxacyclohexadec‐3‐en‐6‐yl) ester, [5R‐(3E, 5R*,6S*,16R*)]‐ (9 CI), CAS No. 122 211‐62−5) each possess eight rotatable bonds, compound **4** results in 19, compound **5** gives rise to 32, and compound **6** results in 57 conformers. Compound **7** (C_16_H_28_O_5_, oxacyclohexadec‐3‐en‐2‐one, 5,6,15‐trihydroxy‐16‐methyl‐, (3E, 5R,6S, 15S,16R)‐ (ACI), CAS No. 2 095 114‐72−8) contains only four rotatable bonds capable of inducing conformational changes and yields six conformers. Compounds **8** and **9** have nine, accompanied by conformational variations in their ring systems, compound **8** (C_20_H_32_O_8_, butanedioic acid, 1‐[(3E, 5R,6S, 15S,16R)−5,15‐dihydroxy‐16‐methyl‐2‐oxooxacyclohexadec‐3‐en‐6‐yl] ester (ACI), CAS No. 2 095 114‐73−9) produces 15, compound **9** (C_20_H_32_O_8_, butanedioic acid, 1‐[(3E, 5R,6S, 16S)‐5‐hydroxy‐16‐(hydroxymethyl)‐2‐oxooxacyclohexadec‐3‐en‐6‐yl] ester (ACI), CAS No. 2 095 114‐74−0) yields 42. In comparison, macrolide **M** (C_41_H_67_NO_15_, Leucomycin V, 3,4^B^‐dipropanoate (9 CI, ACI), CAS No. 35 457‐80−8) exhibits as many as 246 conformers. This indicates that the carbonyl group of the carboxylic acid in compound **2** forms a conjugated system with the hydroxyl group, thereby significantly limiting the diversity of its pharmacologically active conformations. Existing research [49] has demonstrated that conformational restriction not only enhances drug safety but also improves pharmacological activity. Previously, we reported a computational study comparing the structural and physicochemical properties of clinically used drugs and potential candidate compounds for multidrug‐resistant tuberculosis (MDR‐TB) using the M06‐2X/6‐311+G(2d, p) method [50]. The results demonstrated that the novel compound 19c undergoes ring closure at the 3aS position, leading to a significant reduction in pharmacophore conformational flexibility. This finding is consistent with experimental observations that conformational constraints enhance drug safety and therapeutic efficacy.

### 3.2 Geometric Structures

Figure 2 presents the geometric structures of nine novel 16‐membered lactone compounds and midecamycin in vacuum, water, and methanol environments, calculated using the *ω*B97XD/6‐311+G(2d, p) method. The main bond lengths are labeled at their respective positions. Table S1 compares the theoretically calculated bond lengths and angles of compound 1 under vacuum conditions with the experimentally determined values from crystallographic data [4]. Following the substitution of the hydroxyl group in compound **1** with organic acid esters, a notable effect was observed on the O—H bond of the side chain S. Specifically, the hydroxyl O—H bond shortened by approximately 0.008 Å, whereas the carboxyl O—H bond elongated by about 0.015 Å. Upon introduction of a hydroxyl group into compound **3**, significant changes were observed in the C=O and C—O bond lengths of the ester moiety in the side chain, with the C=O bond shortening by approximately 0.002 Å and the C—O bond elongating by about 0.004 Å. This effect was more pronounced in aqueous and methanolic environments compared to the gas phase. When the carbonyl group of **3** was replaced by a hydroxyl group, excluding the ring ester C—O bond, the C=O bond length of compound **6** in the gas phase was approximately 0.005 Å shorter than that of compound **3**, the C—O bond was approximately 0.006 Å longer, and the O—H bond length decreased by about 0.011 Å. Following the substitution of the hydroxyl group in compound **7** with an organic acid ester, both the O—H and C—O bond lengths on the ring of compound **8** in the gas phase were approximately 0.004 Å longer than those in compound **7**. Finally, upon replacing the methyl group of **6** with a methoxy group, the C—O bond length on the ring of compound **9** in the gas phase was approximately 0.003 Å longer than that of compound **6**.

As solvent polarity increases, most bond lengths exhibit only minor variations; however, the C=O and C—O bonds demonstrate more pronounced changes. Upon transitioning from the gas phase to aqueous or methanolic environments, the C=O bond in compounds **3**, **6**, and **9** elongates by approximately 0.005 Å, while the C—O bond shortens by approximately 0.008 Å. In compounds **7** and **8**, the C=O bond lengthens by approximately 0.006 Å, and the C—O bond contracts by approximately 0.01 Å. Overall, the solvent environment exerts a relatively modest influence on bond lengths, and the trends observed in aqueous and methanolic systems are highly consistent.

As presented in Table S1, under the applied theoretical method and basis set, the calculated carbonyl bond lengths exhibit excellent agreement with the experimental values, showing deviations within the range of −0.002 to −0.004 Å. In contrast, the hydroxyl bond lengths show noticeable discrepancies between theoretical and experimental values, with deviations ranging from 0.112 to 0.211 Å, suggesting lower consistency. A comparison of the optimized bond lengths and bond angles with X‐ray crystallographic data reveals that the main bond lengths have an error range of −0.028–0.206 Å, with an average absolute error of 0.049 Å and a relative error of 3.80%. For the central bond angles, the error range is −2.4° to 3.8°, with an average absolute error of 2.000° and a relative error of 0.91%. These results demonstrate that the geometric structural parameters derived from theoretical calculations are highly consistent with the experimental crystallographic data. The minor discrepancies observed are likely due to environmental differences: the experimental data were obtained under solid‐state conditions, whereas the theoretical calculations were performed under gas‐phase assumptions.

### 3.3 IR Spectroscopy

**FIGURE 3:**
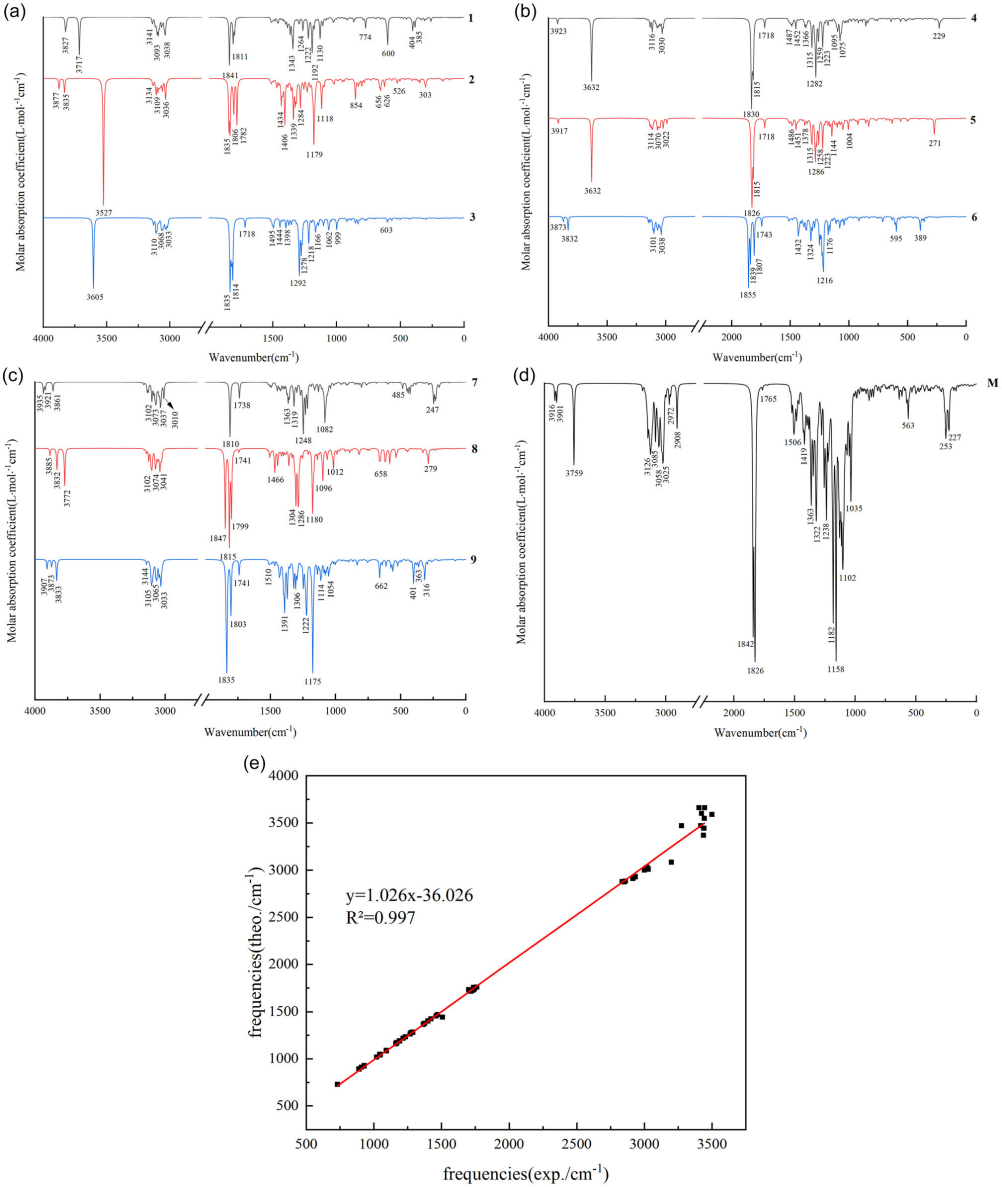
Presented are the infrared absorption spectra of 16‐membered lactone ring compounds exhibiting potential antibacterial activity, derived from secondary metabolites of extremophilic microorganisms, and of midecamycin, calculated using the *ω*B97XD/6‐311+G(2d, p) method in vacuum (a–d), together with linear regression analysis comparing theoretical and experimental vibrational frequencies (e).

Figure 3 displays the vacuum IR absorption spectra of 10 compounds calculated at the *ω*B97XD/6‐311+G(2d, p) theoretical level (Figure 3a–d), along with the linear regression analysis comparing theoretical and experimental vibrational frequencies [4] (Figure 3e). The detailed IR peak assignments based on both theoretical calculations and experimental data are provided in Table S2. As illustrated in Figure 3, the C=O stretching vibration exhibits strong IR absorption, with characteristic peaks predominantly in the 1850–1800 cm^−1^ range. The O–H stretching vibrations are mainly observed within the 3900–3600 cm^−1^ region, while the C–C stretching vibrations on the parent ring are primarily concentrated between 1765 and 1715 cm^−1^. A comparison between compounds **1** and **2** reveals that the substitution of the hydroxyl group with an organic acid ester results in a red shift of approximately 300 cm^−1^ in the O–H stretching vibration of the side‐chain carboxyl group, whereas a blue shift of about 160 cm^−1^ occurs in the O–H stretching vibration of the hydroxyl group on the parent ring. This phenomenon is likely associated with the disruption of pre‐existing hydrogen bonding interactions and the formation of new hydrogen bonds. Further analysis of compounds **3**, **4**, and **5** indicates that the introduction of a hydroxyl group on the parent ring leads to a blue shift of approximately 30 cm^−1^ in the O–H stretching vibration of the carboxyl group, with minimal impact on the C=O stretching frequency. Comparative studies of compounds **6** and **8** demonstrate that the introduction of a hydroxyl group on the parent ring in compound **6** induces a red shift of approximately 5–40 cm^−1^ in the side chain C=O stretching vibration, a blue shift of roughly 8 cm^−1^ in the parent ring C=O stretching vibration, and a red shift of approximately 25 cm^−1^ in the C–C stretching vibration.

As indicated by the results presented in Table S2 and Figure 3e, the *R* ^2^ value of 0.997 exceeds 0.950, demonstrating a high level of correlation between the theoretically calculated IR vibrational frequencies and the experimental data. However, the agreement between theoretical and experimental characteristic absorption peaks is relatively lower in the high‐frequency region (3000–3700 cm^−1^). In contrast, it is notably improved in the low‐frequency region (below 1700 cm^−1^). This discrepancy can be attributed to several factors. First, the theoretical calculations are based on a single‐molecule model under vacuum conditions. In contrast, experimental data are typically obtained from solid‐state powder samples, where various intermolecular interactions are present. Additionally, most compounds were analyzed in chloroform during the experimental measurements. In contrast, solvent effects were not incorporated into the theoretical simulations, leading to more pronounced deviations in the high‐frequency range.

### 3.4 Ultraviolet–Visible Absorption Spectroscopy

**FIGURE 4:**
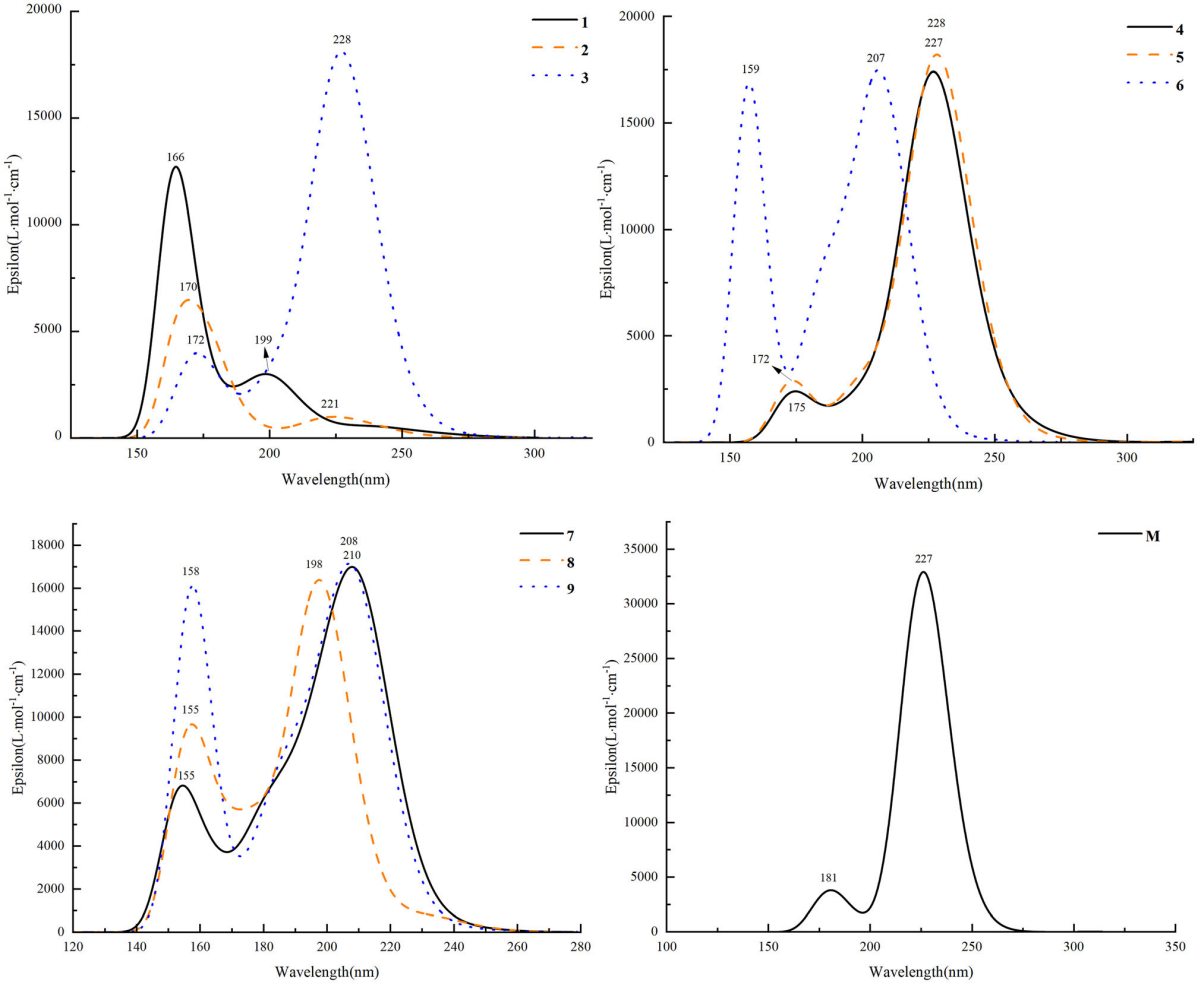
UV–Vis absorption spectra of 16‐membered lactone ring compounds exhibiting potential antibacterial activity, derived from secondary metabolites of extremophilic microorganisms, and of midecamycin, calculated in methanol using the *ω*B97XD/6‐311+G(2d, p) method.

The UV–Vis absorption spectra of 10 macrolide compounds in methanol were computed at the *ω*B97XD/6‐311+G(2d, p) theoretical level. The vertical excitation energies (E), absorption wavelengths (*λ*), oscillator strengths (*f*), and dominant electronic transition configurations are summarized in Table 1. The resulting UV–Vis absorption spectral profiles are presented in Figure 4. The analysis reveals that all 10 compounds exhibit two distinct absorption bands within the 150–230 nm range, corresponding to the far‐UV (150–196 nm) and near‐UV (197–230 nm) regions, both of which originate from *π* → π* electronic transitions. Comparative analysis of compounds **1** and **2**, as well as **7** and **8**, indicates that substitution of the hydroxyl group with an organic acid ester leads to a red shift of approximately 15 nm in the near‐UV absorption band. This shift can be attributed to enhanced conjugation effects induced by enolization or intramolecular condensation, along with the stabilization of the conjugated system through intramolecular hydrogen bonding. Further comparison of compounds **3** and **6** reveals that replacing the carbonyl group with a hydroxyl group results in a blue shift of approximately 14 nm in the far‐UV absorption band, accompanied by a corresponding increase in absorption intensity. Additionally, the near‐UV absorption band exhibits a blue shift of approximately 22 nm, which may be associated with reduced conjugation and the electron‐donating nature of the hydroxyl group. These findings require further experimental validation.

**TABLE 1:** UV–Vis spectral parameters of 16‐membered lactone ring compounds exhibiting potential antibacterial activity, derived from secondary metabolites of extremophilic microorganisms, and of midecamycin, calculated in methanol using the *ω*B97XD/6‐311+G(2d, p) method.

Compd.	E/eV	*λ*/nm	*f*	Configuration (%)		UV transition type
**1**	7.47	166.07	0.0881	H‐1→L + 1^a^	24.1	*Π*→*Π* a
	6.24	198.76	0.0384	H‐2→L	40.1	*Π*→*Π* a
**2**	7.30	169.83	0.0025	H→L + 11	25.7	*Π*→*Π* a
	5.60	221.25	0.0180	H→L + 3	26.8	*Π*→*Π* a
**3**	7.23	171.57	0.0129	H‐10→L	46.1	*Π*→*Π* a
	5.45	227.55	0.4386	H‐5→L	50.6	*Π*→*Π* a
**4**	7.07	175.39	0.0132	H‐14→L	36.7	*Π*→*Π* a
	5.46	227.07	0.4208	H‐5→L	49.3	*Π*→*Π* a
**5**	7.23	171.56	0.0211	H‐14→L	27.5	*Π*→*Π* a
	5.43	228.36	0.4451	H‐6→L	43.7	*Π*→*Π* a
**6**	7.80	158.89	0.1049	H‐4→L + 2	22.4	*Π*→*Π* a
	5.98	207.31	0.3925	H→L	63.1	*Π*→*Π* a
**7**	8.01	154.78	0.0604	H‐3→L + 1	35.1	*Π*→*Π* a
	5.90	210.21	0.3684	H→L	62.3	*Π*→*Π* a
**8**	7.97	155.48	0.0411	H‐2→L + 3	27.3	*Π*→*Π* a
	6.25	198.41	0.3749	H→L	57.3	*Π*→*Π* a
**9**	7.83	158.41	0.1064	H‐2→L	18.5	*Π*→*Π* a
	5.95	208.43	0.3872	H→L	63.6	*Π*→*Π* a
**M**	6.81	182.17	0.0406	H→L + 2	25.8	*Π*→*Π* a
	5.48	226.21	0.8111	H‐1→L	69.1	*Π*→*Π* a

a H, highest occupied molecular orbital; L, lowest unoccupied molecular orbital.

The primary origin of the UV absorption bands is the electronic transition from the highest occupied molecular orbital (HOMO) to the lowest unoccupied molecular orbital (LUMO). Midecamycin (**M**) exhibits absorption maxima at 181 nm and 227 nm. According to Tsuruoka et al. [51], a UV absorption peak near 230 nm was reported, which aligns closely with the theoretical predictions of this study. The UV absorption spectrum of **M** is predominantly attributed to the electronic transition from the HOMO‐1 to the LUMO, with a contribution of approximately 69.1%.

### 3.5 ECD Analysis

**FIGURE 5:**
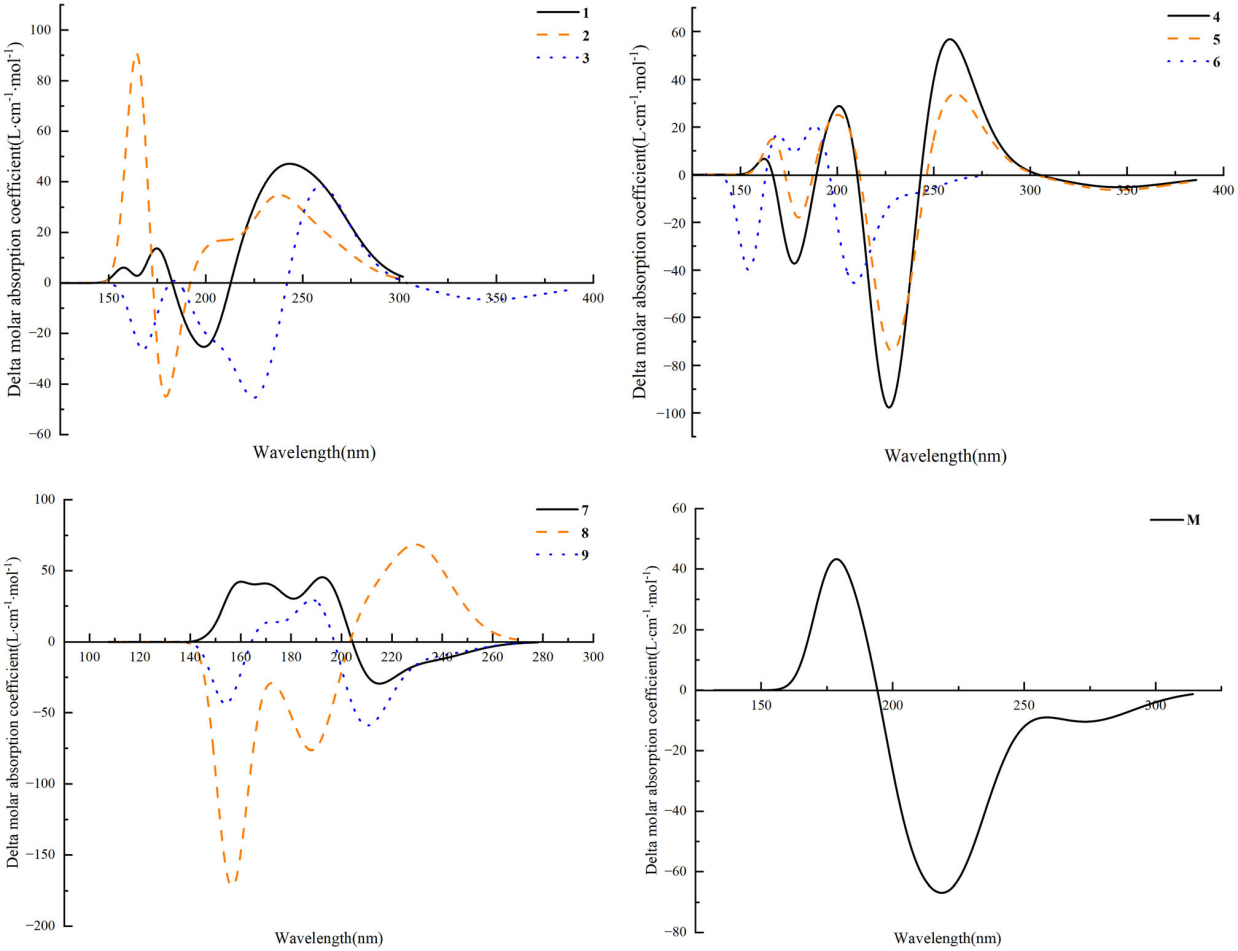
ECD spectra of compounds exhibiting potential antibacterial activity, derived from secondary metabolites of extremophilic microorganisms containing a 16‐membered lactone ring, including midecamycin, were calculated in methanol using the *ω*B97XD/6‐311+G(2d, p) computational method.

The molar absorption coefficients and absorption wavelengths of 16‐membered lactone ring compounds with potential antibacterial activity, derived from extreme microbial secondary metabolites, and of midecamycin in methanol were calculated at the *ω*B97XD/6‐311+G(2d, p) theoretical level. The results are presented in Figure 5. The ECD spectral profiles of compounds **3** and **4**, **5** and **6**, and compound **9** exhibit a high degree of similarity. All 10 compounds possess at least two chiral centers. The substitution of ring hydrogen atoms by hydroxyl groups alters molecular chirality, leading to the reversal of the ECD signal orientations for compounds **6** and **8** at 155 and 188 nm.

### 3.6 ^1^H NMR and ^13^C NMR Characterization Results

Building upon the structural optimization, the ^13^C NMR and ^1^H NMR chemical shifts of nine compounds with potential antibacterial activity, derived from extremophilic microbial secondary metabolites were computed using the GIAO method at the *ω*B97XD/6‐311+G(2d, p) level of theory. The influence of the methanol (CH_3_OH) solvent environment was incorporated through the polarizable continuum model (PCM). At the same computational level, the ^13^C NMR and ^1^H NMR chemical shifts of compound **1** were also calculated, with the solvent effect of chloroform (CHCl_3_) simulated using the PCM approach. To facilitate direct comparison with experimental data, tetramethylsilane (TMS) was used as the reference standard for chemical shift calculations, and both proton and carbon resonance signals were separately scaled to account for the corresponding solvent effects.

**FIGURE 6:**
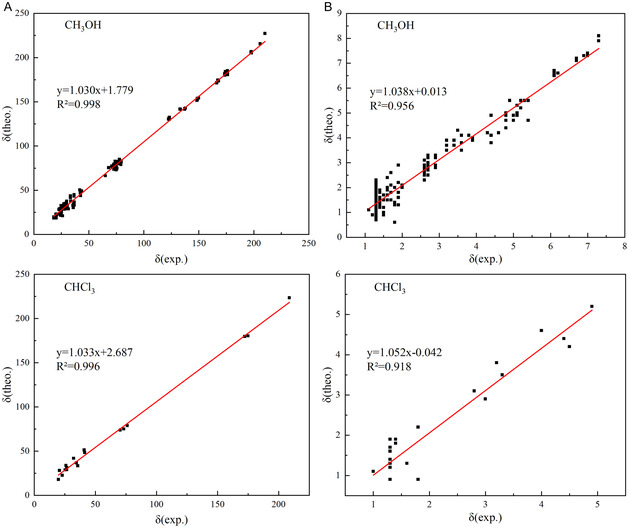
Fitting plots comparing the calculated ^13^C NMR (A) and ^1^H NMR (B) chemical shifts of potential antibacterial compounds from the 16‐membered lactone ring class of secondary metabolites derived from extremophilic microorganisms, obtained using the *ω*B97XD/6‐311+G(2d, p) method in methanol or chloroform, with the corresponding experimental values.

Table S3 presents the theoretically calculated and experimentally measured ^13^C and ^1^H NMR chemical shifts for nine compounds with potential antibacterial activity, derived from extremophilic microbial secondary metabolites, in CH_3_OH and CHCl_3_ environments. In both solvent environments, the ^13^C NMR data reveal significant deviations between the theoretical and experimental values [4] for the carbonyl carbon atoms on the 16‐membered lactone ring, followed by the ester carbon atoms. In the ^1^H NMR data, hydrogen atoms adjacent to carbonyl groups exhibit relatively poor agreement, with those near ester groups showing moderate discrepancies. To evaluate the reliability of the computational method, the root mean square deviation (RMSD) and mean absolute deviation (MAD) between theoretical and experimental NMR data for compounds **1**–**9** in methanol and for compound **1** in chloroform were calculated, with the results presented in Table 2. For ^13^C NMR, the RMSD ranged from 4.21 to 6.28 and the MAD from 3.45 to 5.20 ppm; for ^1^H NMR, the RMSD ranged from 0.29 to 0.41 and the MAD from 0.25 to 0.32 ppm. The results indicate that the prediction accuracy of compound **1** is consistently lower in both methanol (CH_3_OH) and chloroform (CHCl_3_), primarily due to a significant deviation in the predicted chemical shift of C10 (the carbonyl carbon). To further assess the correlation between theoretical calculations and experimental measurements^4^, linear regression analyses were performed. As illustrated in Figure 6, in the CH_3_OH environment, the linear regression equations are *y* = 1.030x + 1.779 (*R* ^2^ = 0.998) for ^13^C NMR and *y* = 1.038x + 0.013 (*R* ^2^ = 0.956) for ^1^H NMR. In the CHCl_3_ environment, the corresponding equations are *y* = 1.033x + 2.687 (*R* ^2^ = 0.996) for ^13^C NMR and *y* = 1.052x – 0.042 (*R* ^2^ = 0.918) for ^1^H NMR. Notably, the CHCl_3_ data originate solely from compound **1**, whereas the CH_3_OH data encompass all nine 16‐membered lactone ring compounds. The regression results indicate that, aside from the ^1^H NMR data of compound **1** in CHCl_3_ (*R* ^2^ = 0.918), all other *R* ^2^ values exceed 0.95, demonstrating that the NMR data obtained using this theoretical method are highly accurate and that the theoretical predictions are in strong agreement with the experimental results.

**TABLE 2:** A comparison between theoretical and experimental ^13^C and ^1^H NMR chemical shift data of 16‐membered lactone ring compounds exhibiting potential antibacterial activity, derived from secondary metabolites of extremophilic microorganisms, in methanol (compounds 1–9) and chloroform (compound 1) [4].

**CH**_**3**_ **OH**		1	2	3	4	5
^13^C NMR	RMSD	6.05	5.30	5.41	5.20	4.86
	MAD	4.96	4.60	4.76	4.29	4.06
^1^H NMR	RMSD	0.38	0.35	0.37	0.41	0.40
	MAD	0.27	0.28	0.29	0.32	0.31

CH_3_OH		**6**	**7**	**8**	**9**	
^13^C NMR	RMSD	4.44	4.43	5.74	4.21	
	MAD	3.78	3.63	5.02	3.45	
^1^H NMR	RMSD	0.32	0.34	0.38	0.29	
	MAD	0.26	0.29	0.32	0.25	

CHCl_3_		**1**				
^13^C NMR	RMSD	6.28				
	MAD	5.20				
^1^H NMR	RMSD	0.37				
	MAD	0.29				

### 3.7 Molecular Electrostatic Potential (ESP) Distribution

**FIGURE 7:**
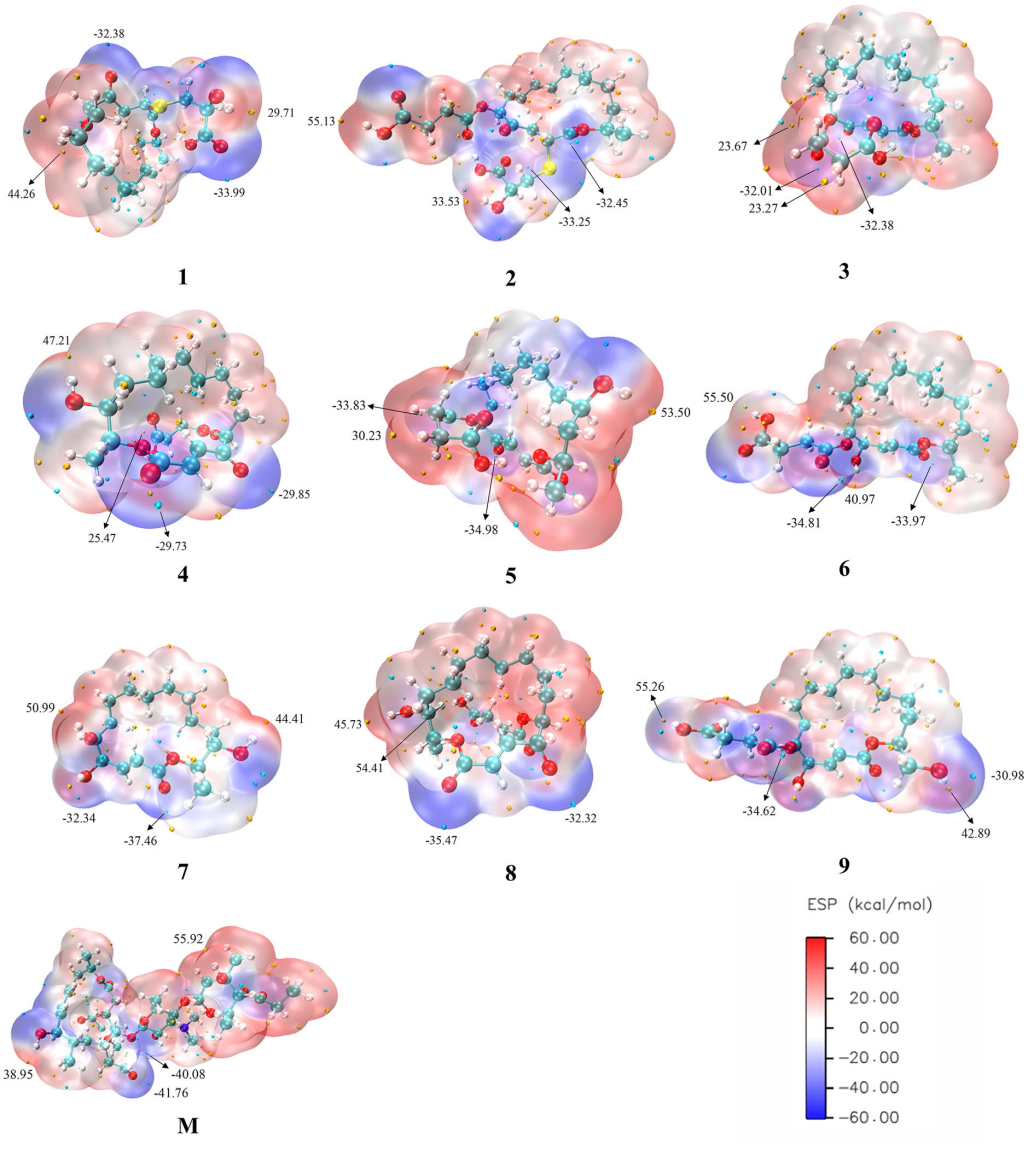
Molecular electrostatic potential maps of compounds exhibiting potential antibacterial activity, derived from secondary metabolites of extremophilic microorganisms containing 16‐membered lactone rings, as well as midecamycin, calculated in the gas phase using the *ω*B97XD/6‐311+G(2d, p) method.

The molecular surface ESP [52, 53, 54] is a key theoretical tool for characterizing molecular reactive regions by identifying nucleophilic and electrophilic reaction sites. ESP is widely employed in the study of molecular interactions and recognition mechanisms, offering insights into the relationship between chemical reactivity and electronic structure. Figure 7 presents the molecular surface ESP distributions of compounds with potential antibacterial activity, including those derived from 16‐membered lactone ring‐containing secondary metabolites of extremophilic microorganisms, as well as midecamycin. On the map, blue regions indicate areas of negative ESP, which are susceptible to nucleophilic attack; red regions represent areas of positive ESP, which are prone to electrophilic attack; and white regions correspond to areas with near‐zero ESP. The yellow spheres denote the ESP maxima (i.e., the regions with the highest positive potential), while the cyan spheres indicate the ESP minima (i.e., the regions with the lowest negative potential).

Based on the analysis of the ESP map, the maxima of ESP are predominantly located near the hydrogen atoms of carboxyl or hydroxyl groups. In contrast, the minima are primarily concentrated around the oxygen atoms of ester or carbonyl groups. In compound **4**, the oxygen atom of the carbonyl group on the parent ring serves as the electrophilic reaction active site, exhibiting an ESP minimum of −29.85 kcal/mol, which is higher than those observed in other compounds (−41.76 to −32.38 kcal/mol), suggesting relatively lower electrophilic reactivity. In compound **3**, the oxygen atom of the side‐chain ester group serves as the nucleophilic reaction center, with an ESP maximum of 23.67 kcal/mol, which is lower than those of other compounds (47.21 to 55.92 kcal/mol), indicating comparatively reduced nucleophilic reactivity.

Furthermore, the carboxylic acid ester oxygen atoms in compounds **1**–**3** exhibit pronounced electrophilic reactivity, with corresponding minimum ESP values of −33.99, −32.25, and −32.38 kcal/mol, respectively. Similarly, the lactone oxygen atoms in compounds **4**–**8** demonstrate electrophilic reactivity, with minimum values of −29.73, −34.98, −33.97, −37.46, and −35.47 kcal/mol, respectively. Compared to compounds **1**–**3**, compounds **4**–**8**, which possess hydroxyl groups on the parent ring and lack sulfur‐containing side chains, exhibit enhanced electrophilic reactivity near the lactone oxygen atoms. Compounds **1**, **4**, **5**, and **7** exhibit the highest maximum ESPs near the hydroxyl hydrogen atoms at 44.26, 47.21, 53.50, and 50.99 kcal/mol, respectively. Analysis suggests that the hydroxyl groups in these compounds are directly attached to the parent ring, thereby enhancing their nucleophilic reactivity.

### 3.8 Frontier Molecular Orbital (FMO) Analysis

FMO analysis constitutes a fundamental theoretical framework within molecular orbital theory. Evaluation of the HOMO and LUMO enables prediction of a molecule's electron‐donating and electron‐accepting capacities in chemical reactions. Specifically, the HOMO reflects electron‐donating ability, whereas the LUMO indicates electron‐accepting propensity. A larger HOMO–LUMO energy gap indicates greater thermodynamic stability and lower chemical reactivity. For bioactive molecules, this property directly influences their interaction mechanisms with biological receptors and the stability of metabolic processes. The orbital energy levels at the receptor's active site may align with those of the ligand's HOMO or LUMO orbitals, or in cases, where electron transfer is suppressed, molecular binding can occur via noncovalent interactions. As reported by Abrosimov et al. [55], bioactive molecules—such as secondary metabolites—typically exhibit smaller HOMO–LUMO energy gaps, which enhance reactivity and functional diversity; conversely, larger energy gaps may constrain their adaptability in rapidly evolving biological environments. The FMO energy gaps of compounds exhibiting potential antibacterial activity, along with midecamycin, derived from the 16‐membered lactone ring class of secondary metabolites produced by extremophilic microorganisms, range from 8.69 to 10.32 eV (Figure 8). Compound **3** exhibits the smallest HOMO–LUMO energy gap (Δ*ε* = 8.69 eV) among the series, suggesting a higher electron‐donating capacity and enhanced molecular reactivity. In contrast, compound **8** exhibits a relatively larger orbital energy gap (Δ*ε *= 9.54 eV) and reduced electron mobility, indicating a diminished electron‐accepting ability, greater molecular stability, and lower reactivity.

FIGURE 8Frontier molecular orbital distribution diagrams of compounds exhibiting potential antibacterial activity, derived from secondary metabolites of extremophilic microorganisms containing a 16‐membered lactone ring, as well as midecamycin.

**Figure:**
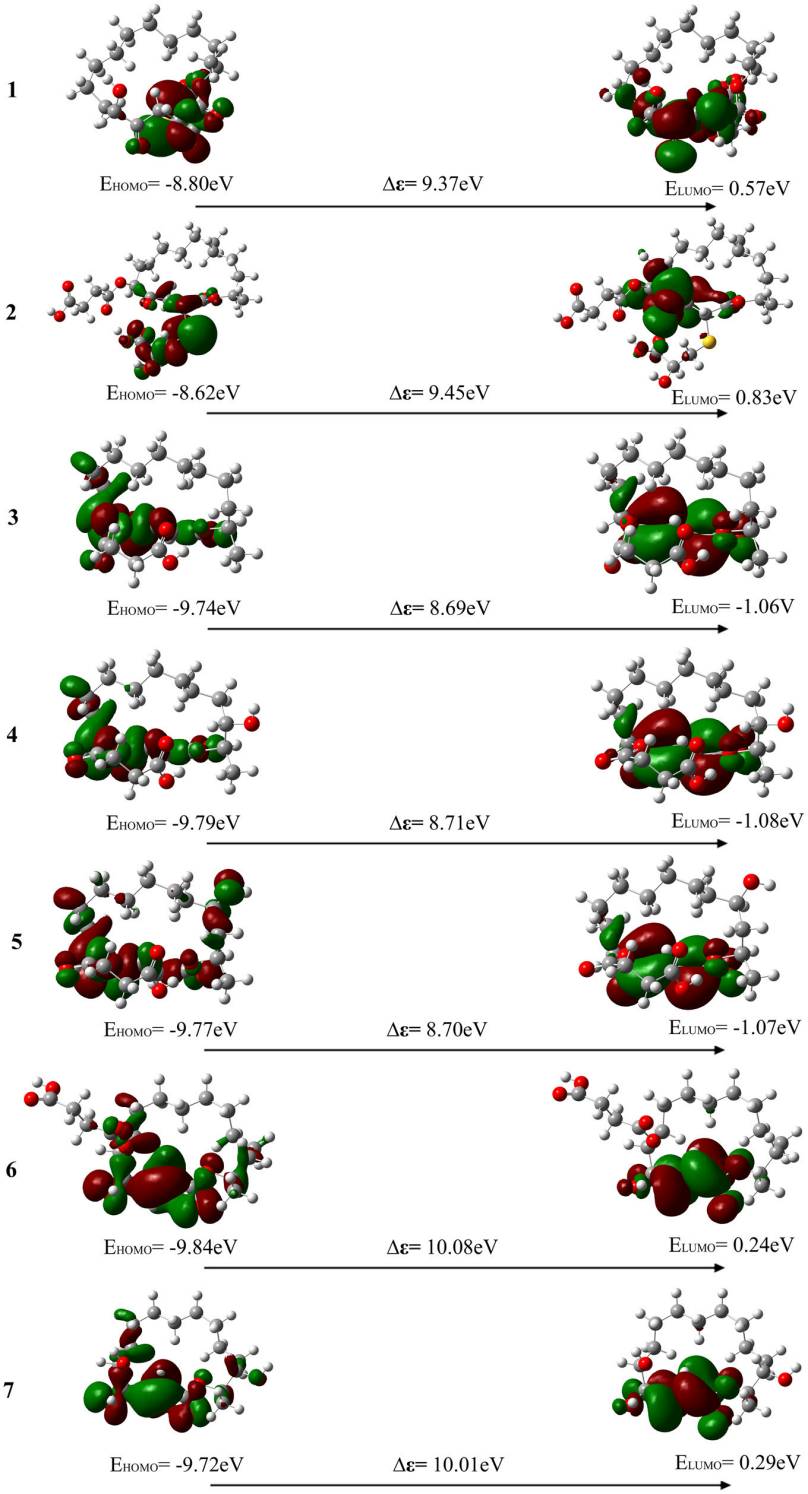

**Figure:**
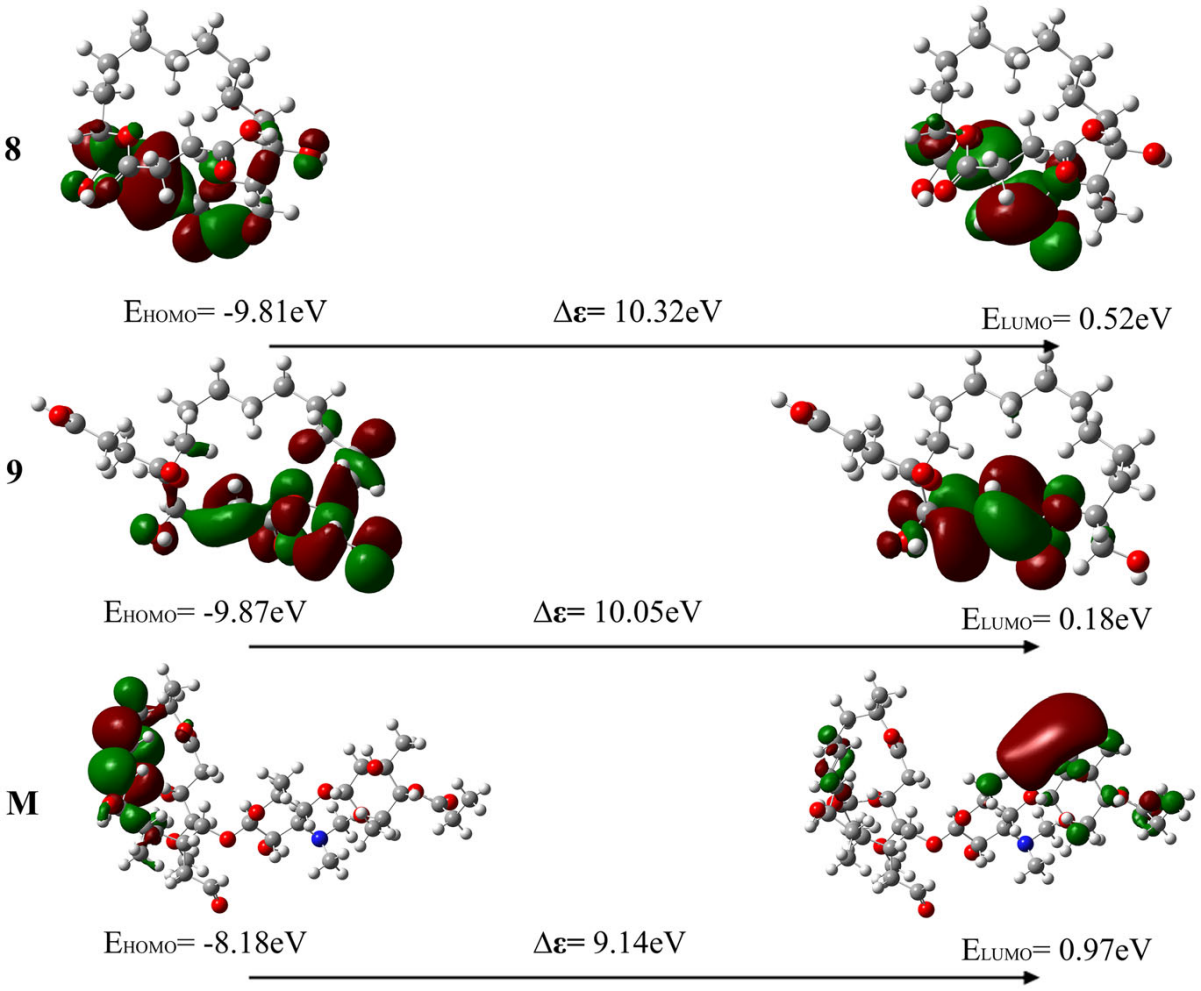

The HOMO and LUMO of compounds **1** and **2** are predominantly localized within the sulfur‐containing side‐chain regions. In contrast, the molecular orbitals of compounds **3** to **9** are primarily delocalized across the conjugated lactone ring system. In midecamycin, the HOMO is mainly distributed over the conjugated lactone region, whereas the LUMO is predominantly localized in the pyran ring moiety distal to the parent ring. Further analysis of electron delocalization patterns across the molecular orbitals of all 10 compounds reveals that compounds **3** and **4**, as well as compounds **6** and **7**, exhibit highly similar HOMO and LUMO distribution profiles. These findings provide valuable insights into the electronic structure and molecular stability of this compound class.

### 3.9 Global Reactivity Descriptors

Table 3 presents the global reactivity parameters of the 10 studied compounds. Using midecamycin (**M**) as a reference standard, a comparative analysis was conducted of its reactivity descriptors with those of potential antibacterial compounds derived from 16‐membered lactone ring‐containing secondary metabolites of extremophilic microorganisms. The results indicate that compounds **3**, **4**, **5**, **6**, and **9** exhibit relatively low chemical potentials (*μ*), with compound **4** displaying the lowest *μ* value, suggesting superior thermodynamic stability with respect to global hardness (*η*), compounds **1**, **6**, **7**, **8**, and **9** exhibit higher *η* values, indicating greater resistance to electron cloud distortion under external perturbations and enhanced overall system stability. The electrophilicity index (*ω*) reflects a compound's ability to accept electrons. As shown in Table 3, compounds **3**, **4**, **5**, **6**, and **9** possess strong electron‐accepting capabilities and exhibit high chemical reactivity, with compound 4 displaying the highest electrophilicity index. Compound **2** exhibits global reactivity parameters that are highly similar to those of midecamycin. A comprehensive evaluation reveals that compounds **6** and **9** not only possess favorable structural stability but also demonstrate significant chemical reactivity.

**TABLE 3:** Global reactivity descriptors (in eV) of macrolide ring‐containing secondary metabolites with potential antibacterial activity derived from extremophiles, and of midecamycin (with S expressed in eV^−1^).

Compd.	*IP*	*EA*	*χ*	*η*	*μ*	*ω*	*S*
**1**	9.0220	−0.9364	4.0428	9.9584	−4.0428	0.8206	0.1004
**2**	8.3138	−0.6909	3.8114	9.0046	−3.8114	0.8066	0.1111
**3**	9.4188	1.1032	5.2610	8.3156	−5.2610	1.6643	0.1203
**4**	9.5380	1.1546	5.3463	8.3835	−5.3463	1.7047	0.1193
**5**	9.5241	1.1518	5.3379	8.3723	−5.3379	1.7016	0.1194
**6**	9.7404	−0.1349	4.8027	9.8753	−4.8027	1.1679	0.1013
**7**	9.5117	−0.3967	4.5575	9.9084	−4.5575	1.0481	0.1009
**8**	9.7181	−0.3390	4.6895	10.0571	−4.6895	1.0933	0.0994
**9**	9.7164	−0.0478	4.8343	9.7642	−4.8343	1.1967	0.1024
**M**	7.7974	−0.7771	3.5101	8.5745	−3.5101	0.7185	0.1166

### 3.10 Condensed Fukui Functions

In addition to global reactivity descriptors, the condensed Fukui functions (*f* ^+^, *f* ^−^, *f* ^0^) of individual atoms play a crucial role in elucidating molecular reaction mechanisms. To further identify the reactive sites within the drug molecules, this study conducted a systematic analysis of the condensed Fukui functions (*f* ^+^, *f* ^−^, *f* ^0^), electrophilicity indices (*ω* ^+^, *ω* ^−^), and softness parameters (s^+^, s^−^) for the 10 compounds under investigation. The results indicate that the most electrophilic reactive site in midecamycin is O53, which aligns with previous literature [56]. Moreover, the C4 position is predicted to be a potential reactive site, with a slight deviation from the C5 position reported in the references [56, 57]. As presented in Table 4, the 17th sulfur atom in compounds **1** and **2**, the 28th oxygen atom in compounds **4** and **5**, and the 27th oxygen atom in compound **3** exhibit the highest *f* ^−^, *ω* ^−^, and s^−^ values, suggesting that these sulfur atoms and ring carbonyl oxygen atoms are particularly susceptible to electrophilic attack. These findings demonstrate that the reactive sites of the nine natural products differ significantly from those of midecamycin, further supporting the hypothesis that the mode of action of Berkeley Lake lactone ring‐containing compounds differs from that of established macrolide antibacterial agents [4].

**TABLE 4:** Local reactivity descriptors of potential antibacterial compounds containing macrolide ring structures derived from extremophilic microorganisms, and of midecamycin.

Compd.	Atom	***f*** ^**−**^	***f*** ^***+***^	***f*** ^**0**^	***ω*** ^**−**^	***ω*** ^**+**^	**s** ^**−**^	**s** ^**+**^
**1**	C20	−0.4152	0.4386	0.0117	0.3600	−0.2383	−1.1344	1.1986
	S17	0.4380	0.0076	0.2228	0.0062	0.2514	1.1967	0.0206
**2**	C10	0.0027	0.1961	0.0994	0.1582	0.0021	0.0080	0.5926
	S17	0.4142	0.0399	0.2271	0.0322	0.3205	1.2517	0.1206
**3**	O27	0.2714	0.1250	0.1982	0.2080	−0.1163	0.8880	0.4090
	C2	0.0777	0.0951	0.0864	0.1582	−0.0333	0.2542	0.3111
**4**	O28	0.2735	0.1282	0.2009	0.2185	−0.1495	0.8879	0.4160
	C4	0.0666	0.1190	0.0928	0.2028	−0.0364	0.2161	0.3862
**5**	O28	0.2723	0.1276	0.2000	0.2172	−0.1445	0.8849	0.4148
	C4	0.0663	0.1183	0.0923	0.2013	−0.0352	0.2156	0.3844
**6**	C3	0.1172	0.1425	0.1299	0.1664	−0.0813	0.3230	0.3926
	C4	0.1389	0.1113	0.1251	0.1300	−0.0963	0.3828	0.3067
**7**	C3	0.1191	0.1450	0.1320	0.1520	−0.0499	0.3270	0.3982
	C4	0.1521	0.1069	0.1295	0.1120	−0.0637	0.4177	0.2935
**8**	C3	0.1356	0.1431	0.1393	0.1564	−0.0916	0.3669	0.3871
	C4	0.1531	0.1051	0.1291	0.1149	−0.1033	0.4141	0.2844
**9**	C3	0.0722	0.1433	0.1078	0.1715	−0.0433	0.2013	0.3995
	O18	0.1068	0.0205	0.0637	0.0245	−0.0641	0.2977	0.0571
**M**	O53	−0.0015	−0.0034	−0.0025	−0.0024	−0.0017	−0.0049	−0.0107
	C4	0.1454	0.0022	0.0738	0.0016	0.1623	0.4614	0.0071

### 3.11 PK and Toxicity Evaluation

Pharmacological studies have shown that numerous compounds with potential therapeutic effects fail to progress beyond the early stages of development due to limitations in safety, efficacy, and PK profiles. ACD/Labs Percepta is a computational tool based on quantitative structure–activity relationship (QSAR) models, which is widely used to predict key properties related to absorption, distribution, metabolism, excretion, toxicity, and physicochemical behavior of chemical entities. As presented in Table 5, out of the 10 investigated compounds, all except midecamycin conform to Lipinski's Rule of Five [58], exhibit favorable drug‐like properties and demonstrate generally good aqueous solubility.

**TABLE 5:** Drugability evaluation of potential antibacterial compounds containing macrolide ring structures derived from extremophilic microorganisms, including midecamycin.

**Compd.** a	***M***_**w**_	HBD	HBA	TPSA	Log(BCF)	ST	Density	Polarizability	lgP	Lipinski	Solubility
**1**	404.52	3	7	146.43	2.09	55.07	1.23	40.67	2.59	Good	Soluble
**2**	504.59	3	10	189.80	2.47	58.50	1.29	48.80	2.96	Moderate	Soluble
**3**	382.45	1	7	106.97	1.91	46.64	1.16	38.83	2.93	Good	Soluble
**4**	398.45	2	8	127.20	0.72	51.25	1.21	39.43	1.57	Good	Soluble
**5**	398.45	2	8	127.20	0.59	51.25	1.21	39.43	1.40	Good	Soluble
**6**	384.46	2	7	110.13	2.25	48.17	1.16	39.39	3.37	Good	Soluble
**7**	300.39	3	5	86.99	1.20	40.99	1.09	31.71	1.47	Good	Soluble
**8**	400.46	3	8	130.36	1.06	52.88	1.22	39.99	1.85	Good	Soluble
**9**	400.46	3	8	130.36	1.09	53.77	1.22	40.00	1.78	Good	Soluble
**M**	813.97	3	16	206.05	2.66	50.83	1.22	82.61	2.89	Bad	Soluble

a *M*_w_, molecular weight; HBD, No. of hydrogen‐bond donors; HBA, No. of hydrogen‐bond acceptors; TPSA, topological molecular polar surface area; Log(BCF), bioconcentration factor; ST, surface tension; lgP, fat‐water partition coefficient.

PK is the study of the dynamic behavior of small‐molecule drug candidates in the body and is a critical parameter for assessing their drug‐like properties [59, 60]. Predicted PK profiles for the 10 investigated compounds are summarized in Table 6. Caco‐2 cells, derived from human colon carcinoma, constitute a well‐established in vitro model widely employed to investigate drug transport mechanisms and as a predictive tool for intestinal absorption [61]. According to the Caco‐2 cell data, compounds **3**, **6**, and **7**, along with midecamycin (**M**), demonstrate favorable absorption characteristics. Plasma protein binding (PPB) is a key PK factor that influences drug half‐life, clearance, and tissue distribution [62]. As presented in Table 6, all 10 compounds exhibit either moderate or high PPB. Hepatic metabolic enzymes, including CYP1A2, CYP2C9, CYP2C19, CYP2D6, and CYP3A4, are responsible for the biotransformation of over 80% of exogenous substances [63]. Predictive analysis suggests that these compounds typically do not exhibit significant inhibitory effects on the major cytochrome P450 isoforms. Among the 10 compounds, only compound **7** shows notable blood–brain barrier (BBB) permeability, with higher values correlating with greater permeability. The remaining compounds show limited BBB penetration and are therefore expected to achieve low concentrations in the central nervous system (CNS). Regarding human intestinal absorption (HIA), compound **2** exhibits relatively poor absorption.

**TABLE 6:** PK parameters of potential antibacterial compounds containing macrolide ring structures derived from extremophilic microorganisms, as predicted by ACD/Percepta software, including midecamycin.

Compd.	Absorption			Distribution		Metabolism						Toxicity	
	P‐gp substrates	Caco‐2	HIA	PPB	CNS	CYP1A2 inhibitor	CYP2C9 inhibitor	CYP2C19 inhibitor	CYP2D6 inhibitor	CYP3A4 inhibitor	Metabolic stability	Ames	hERG
**1**	0.37	4.50	89.00	93.00	−4.55	0.27	0.31	0.36	0.22	0.20	0.51	0.40	0.37
**2**	0.24	0.80	3.00	94.00	−4.79	0.25	0.33	0.32	0.20	0.12	0.49	0.28	0.30
**3**	0.43	38.50	100.00	90.00	−3.86	0.33	0.43	0.45	0.33	0.27	0.59	0.29	0.36
**4**	0.44	3.10	87.00	85.00	−4.65	0.33	0.37	0.41	0.35	0.26	0.57	0.33	0.34
**5**	0.43	2.40	80.00	81.00	−4.61	0.33	0.36	0.44	0.34	0.25	0.57	0.32	0.34
**6**	0.42	24.50	100.00	89.00	−3.79	0.31	0.32	0.38	0.34	0.22	0.56	0.30	0.34
**7**	0.38	45.90	100.00	75.00	−2.77	0.18	0.23	0.29	0.24	0.28	0.53	0.36	0.31
**8**	0.41	1.80	79.00	78.00	−4.52	0.25	0.31	0.33	0.32	0.26	0.55	0.31	0.41
**9**	0.42	1.60	75.00	74.00	−4.51	0.27	0.34	0.35	0.32	0.26	0.52	0.32	0.39
**M**	0.74	90.10	100.00	44.00	−4.28	0.01	0.01	0.12	0.03	0.53	0.60	0.30	0.31

Among the 18 freely accessible web‐based platforms for predicting physicochemical properties and PK parameters, ADMETLab offers the most comprehensive set of predictive functionalities and demonstrates relatively high accuracy [64]. ADMETLab 3.0 (https://admetmesh.scbdd.com/) represents the latest upgraded version, featuring significantly enhanced capabilities and broader data coverage compared to version 2.0 [65]. Therefore, this study employed ADMETLab 3.0 to predict the ADMET profiles of 10 16‐membered lactone compounds. The detailed results are presented in Table 7. According to the predictions, compound **8** exhibited the highest HIA, followed by compounds **7** and **4**, whereas compound **3** showed the weakest absorption potential. None of the compounds demonstrated inhibitory activity against CYP1A2, CYP2C19, CYP2C9, CYP2D6, CYP3A4, CYP2B6, or CYP2C8 enzymes, nor were they identified as hERG channel inhibitors. Compounds **8** and **9** displayed low Caco‐2 cell permeability, suggesting limited intestinal absorption. Furthermore, all compounds were predicted to be nonsubstrates of P‐glycoprotein (P‐gp). Drugs with high PPB typically exhibit a lower therapeutic index; among the studied compounds, compounds **2** and **3** showed binding rates exceeding 90%. In terms of hepatotoxicity, compounds **1** and **2** were predicted to be the most toxic, whereas compounds **6**, **9**, and midecamycin (**M**) exhibited the lowest hepatotoxic potential. Nephrotoxicity and ototoxicity predictions indicated that midecamycin and compound **2** posed the highest risk of kidney damage, followed by compound **1**. No significant genotoxicity was observed for any of the compounds. Regarding half‐life, midecamycin exhibited the most extended duration, followed by compounds **7** and **8**. Compound **7** demonstrated the highest metabolic stability in human liver microsomes, while compounds **1**, **3**, **4**, and midecamycin showed relatively poor stability, with compound **1** being the least stable (instability probability of 96.3%). Cross‐validation of PK predictions from two independent platforms further confirmed the low Caco‐2 permeability of compounds 8 and 9, as well as the potential for high PPB in compounds **2** and **3**, which could reduce their therapeutic index. Hepatic metabolic stability is a critical parameter in drug development [66], and compound **7** exhibited the most favorable stability profile in human liver microsome assays.

**TABLE 7:** ADMET profiles of potential antibacterial compounds containing 16‐membered macrolide rings derived from extremophilic microbial secondary metabolites, as predicted by ADMETLab 3.0, including midecamycin.

Compd.	Absorption			Distribution	Toxicity				
	Caco‐2 permeability	P‐gp substrates	HIA	PPB	hERG blockers	Human hep atotoxicity	Drug‐induced nephrotoxicity	Ototoxicity	Genotoxicity
**1**	−5.107	0.005	0.082	75.235	0.057	0.427	0.767	0.744	0.003
**2**	−5.240	0.000	0.158	91.866	0.009	0.451	0.921	0.870	0.004
**3**	−5.021	0.000	0.025	94.991	0.025	0.223	0.296	0.465	0.000
**4**	−5.293	0.000	0.349	80.536	0.020	0.253	0.355	0.629	0.003
**5**	−5.187	0.000	0.279	83.043	0.014	0.345	0.574	0.597	0.002
**6**	−5.128	0.000	0.064	86.407	0.033	0.172	0.501	0.474	0.000
**7**	−5.297	0.093	0.388	57.661	0.132	0.237	0.375	0.606	0.000
**8**	−5.520	0.005	0.591	69.216	0.025	0.215	0.599	0.627	0.000
**9**	−5.518	0.000	0.171	78.425	0.019	0.158	0.581	0.678	0.000
**M**	−5.454	0.000	0.191	26.962	0.039	0.181	0.949	0.962	0.120

Compd.	Excretion	Metabolism							
	T _1/2_	CYP1A2 Inhibitor	CYP2C19 Inhibitor	CYP2C9 Inhibitor	CYP2D6 Inhibitor	CYP3A4 Inhibitor	CYP2B6 Inhibitor	CYP2C8 Inhibitor	HLM Stability
**1**	1.393	0.000	0.000	0.000	0.000	0.000	0.007	0.035	0.963
**2**	1.023	0.000	0.000	0.000	0.000	0.000	0.000	0.000	0.246
**3**	0.791	0.000	0.000	0.000	0.000	0.000	0.028	0.011	0.835
**4**	1.315	0.000	0.000	0.000	0.000	0.000	0.000	0.007	0.876
**5**	1.324	0.000	0.000	0.000	0.000	0.000	0.000	0.176	0.092
**6**	0.960	0.000	0.000	0.000	0.000	0.000	0.000	0.000	0.083
**7**	2.168	0.000	0.000	0.000	0.000	0.000	0.083	0.008	0.021
**8**	1.534	0.000	0.000	0.000	0.000	0.000	0.000	0.000	0.099
**9**	1.125	0.000	0.000	0.000	0.000	0.000	0.004	0.000	0.224
**M**	3.028	0.000	0.000	0.000	0.000	0.001	0.000	0.000	0.786

The acute oral toxicity (LD_50_), carcinogenicity, and mutagenicity of macidomycin and nine potential antibacterial 16‐membered ring lactone compounds were assessed using the toxicity prediction platform ProTox 3.0. The results are presented in Table 8. As shown in Table 8, compounds **1**, **2**, **3**, and **7** are classified as toxicity class 5, with predicted LD_50_ values of 5000 mg/kg; compounds **4**, **5**, and **M** belong to toxicity class 4, with predicted LD_50_ values of 1890 mg/kg for compounds **4** and **5**, and 1000 mg/kg for compound **M**; compounds **6**, **8**, and **9** are assigned to toxicity class 2, with predicted LD_50_ values of 7 mg/kg. Furthermore, all 10 compounds analyzed in this study exhibited pessimistic predictions for both carcinogenicity and mutagenicity, indicating the absence of biological activity associated with these endpoints.

**TABLE 8:** Prediction results of the artificial intelligence toxicity prediction software.

Compd.	**Predicted LD**_**50**_	Predicted toxicity class	Average similarity	Prediction accuracy	Carcinogenicity	Mutagenicity
**1**	5000 mg/kg	5	59.95%	67.38%	0.68/Inactive	0.88/Inactive
**2**	5000 mg/kg	5	56.49%	67.38%	0.69/Inactive	0.87/Inactive
**3**	5000 mg/kg	5	73.28%	69.26%	0.68/Inactive	0.88/Inactive
**4**	1890 mg/kg	4	71.91%	69.26%	0.70/Inactive	0.85/Inactive
**5**	1890 mg/kg	4	72.17%	69.26%	0.70/Inactive	0.88/Inactive
**6**	7 mg/kg	2	76.41%	69.26%	0.69/Inactive	0.90/Inactive
**7**	5000 mg/kg	5	75.52%	69.26%	0.66/Inactive	0.83/Inactive
**8**	7 mg/kg	2	76.80%	69.26%	0.70/Inactive	0.85/Inactive
**9**	7 mg/kg	2	76.80%	69.26%	0.77/Inactive	0.81/Inactive
**M**	1000 mg/kg	4	100.00%	100.00%	0.91/Inactive	0.86/Inactive

### 3.12 Molecular Docking

Molecular docking is a computational method used to predict the most probable binding conformation between a ligand and a protein. It is widely employed in drug discovery and structural biology, serving as a powerful tool in these fields. In this study, molecular docking was performed using the 50S ribosomal subunit from *Streptomyces* bacterium as the target. The high‐resolution crystal structure of this ribosome was retrieved from the Protein Data Bank (PDB, https://www.rcsb.org) under accession code 7A18.

The protein structure was preprocessed using PyMOL, with removal of nonessential crystalline water molecules, nontarget ligands, and redundant protein chains to enhance the computational efficiency of molecular docking. Gasteiger partial charges were subsequently assigned to the receptor protein using AutoDock Vina. Ligand structures were drawn in ChemDraw, exported in mol2 format, and directly prepared for docking using AutoDock Vina. The docking search space was defined based on key residues reported in the literature—U2590, C2589, U2588, and G761—with a center coordinate of *x*: 47.016, *y*: 132.623, and *z*: 128.721. The grid box dimensions were set to ensure full inclusion of all specified residues [26, 27]. For each docking run, AutoDock Vina identified the protein–ligand complex conformation with the lowest binding free energy, which was retained as the optimal docking pose. Molecular interactions within the resulting complexes were then analyzed using the PLIP platform and LigPlot+ software. All structural figures were generated using PyMOL. Notably, residue A2041 (Ec2058) serves as a critical binding site, forming a hydrophobic pocket that accommodates the drug molecule and thereby influences its biological activity.

**FIGURE 9:**
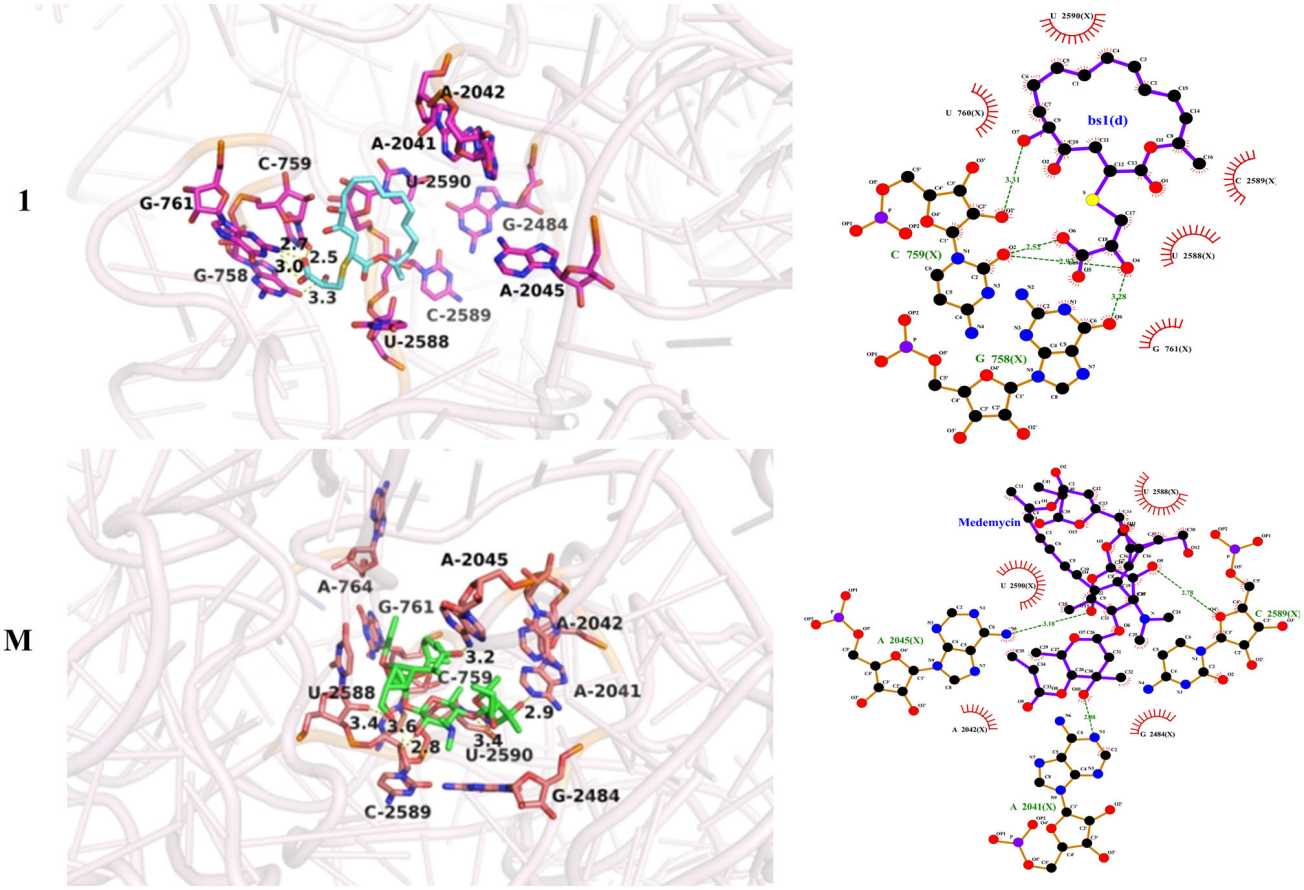
Schematic representation of the two‐dimensional and three‐dimensional molecular docking interaction between compound **1**, midecamycin (**M**), and the 7A18.

Molecular docking analysis was subsequently performed to evaluate the potential for specific binding of compound **1**, which exhibits the most potent antibacterial activity [4] and **M** within this region. The results revealed that macrolide **M** formed a stable interaction with the 7A18, with a binding free energy of −9.21 kcal/mol, indicating strong binding affinity. In contrast, compound **1** exhibited a binding free energy of 1.10 kcal/mol, suggesting weak binding capacity and indicating that 7A18 is not its primary target. Consequently, the antibacterial mechanism of compound **1** differs from that of conventional macrolide antibiotics. This finding is consistent with previous research by Stierle et al. [4]. The detailed two‐dimensional and three‐dimensional binding interactions between the ligands and the 7A18 are presented in Figure 9. As shown in Figure 9, compound **1** binds to a binding pocket of the 7A18 formed by residues U760, U2590, C2589, U2588, G761, G758, and C759. It forms hydrogen bonds with G758 and C759, while engaging primarily in hydrophobic interactions with U760, U2590, C2589, U2588, and G761. Similarly, compound **M** binds to a distinct binding pocket of the 7A18, composed of residues A2042, U2590, U2588, G2482, A2041, A2045, and C2589. **M** establishes hydrogen bonds with A2041, A2045, and C2589, and predominantly interacts with A2042, U2590, U2588, and G2482 via hydrophobic interactions.

### 3.13 MD Simulations

Molecular docking captures only a static snapshot of the ligand–receptor interaction, whereas MD simulation enables dynamic characterization of intermolecular interactions throughout the simulation trajectory. MD simulation results indicate that, overall, small molecules exhibit weak binding affinity toward RNA. In the **M** complex, the ligand gradually dissociates from the initial binding site after 50 ns of simulation and subsequently rebinds to an alternative location. In contrast, the small molecule in complex **1** maintains a more stable binding mode throughout the simulation.

**FIGURE 10:**
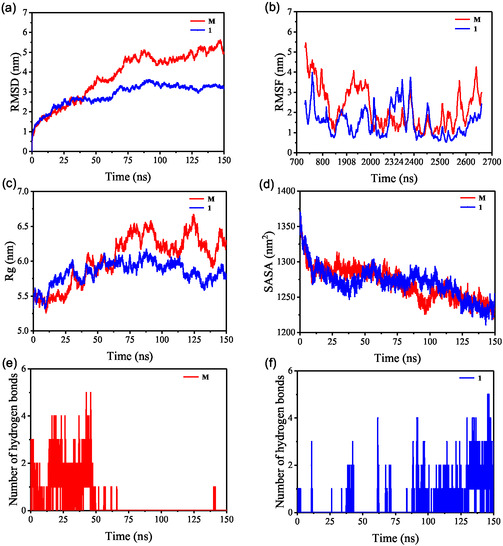
Variation in (a) RMSD, (b) RMSF, (c) Rg, and (d) SASA for **M** and **1**, along with the number of hydrogen bonding interactions between protein and ligand in (e) **M** and (f) **1** during 150 ns MD simulations.

RMSD (Figure 10a) is a widely used metric for assessing the conformational stability of RNA and ligands, as well as for quantifying atomic displacements relative to their initial positions. A smaller RMSD fluctuation range indicates greater conformational stability of the system. In this study, RMSD was employed to evaluate the equilibration of the simulation systems. As shown in Figure 10a, the **M** complex reached equilibrium after 90 ns and exhibited fluctuations within a 5 nm range. In contrast, the **1** complex also achieved equilibrium by 90 ns but displayed a narrower fluctuation range of approximately 3 nm. Compared with the M complex, the 1 complex showed reduced RMSD fluctuations, indicating enhanced structural stability.

Root mean square fluctuation (RMSF) quantifies the deviation of each RNA residue from its time‐averaged position during the simulation. It serves as a reliable indicator of local flexibility within the RNA structure. As shown in Figure 10b, RMSF analysis reveals distinct dynamic profiles across different residues. The radius of gyration (Rg) is a key metric for assessing the structural compactness and stability of RNA. It is commonly used to characterize conformational changes induced by small‐molecule binding. Rg is defined as the root–mean–square distance from the center of mass of all RNA atoms to the overall molecular centroid. As a complementary parameter to RMSD, Rg provides insights into global structural changes. As illustrated in Figure 10c, throughout the 0–150 ns simulation period, both the **M** complex and the **1** complex exhibit a clear trend of structural expansion, with progressively increasing Rg values; notably, the expansion is more pronounced in the **M** complex.

Solvent‐accessible surface area (SASA) is a key metric for assessing the extent of solvent exposure of amino acid residues within a macromolecular system. In general, RNA hydrophobic regions tend to be buried within compactly folded structures, and elevated SASA values indicate structural unfolding or expansion. During the simulation, both the **M** complex and the **1** complex exhibited a pronounced trend toward structural contraction, as evidenced by a progressive decrease in SASA values followed by stabilization. This behavior suggests that the hydrophobic regions gradually reorganized toward the molecular core and became increasingly shielded from the solvent, reflecting enhanced structural compactness.

During the simulation, the number of hydrogen bonds between RNA and the ligand in the **M** complex fluctuated between 0 and 5. After 50 ns, the small molecule dissociated from the RNA binding site, resulting in a complete loss of hydrogen bonds. In contrast, the **1** complex maintained a similar range of 0–5 hydrogen bonds throughout the simulation but exhibited greater stability in hydrogen‐bond interactions. Binding free energies were calculated using the gmx_MMPBSA method. The **M** complex displayed a binding energy of Δ*G* = −14.51 kJ/mol, indicative of weak binding affinity. In comparison, the **1** complex had a binding energy of *G* = −30.76 kJ/mol, indicating moderate binding strength. These results suggest that the **1** complex forms a more stable and energetically favorable complex with the RNA.

## 4 Conclusion

This study presents a comprehensive computational investigation of ten 16‐membered lactone ring compounds, including secondary metabolites derived from extremophilic microorganisms. Geometric structures, spectroscopic properties, and molecular ESPs were calculated using the *ω*B97XD functional with the 6‐311+G(2d, p) basis set. Reactive sites were predicted employing CDFT, while ADME/Tox profiles were assessed using ACD/Labs Percepta and ADMETLab 3.0. Key findings indicate that the conjugated carboxyl‐hydroxyl system in compound **2** restricts conformational flexibility, thereby reducing the number of bioactive conformers. Optimized structural parameters exhibit strong agreement with experimental data, with maximum deviations of 3.80% for bond lengths and 0.91% for bond angles. Theoretical predictions for IR vibrational modes and NMR chemical shifts demonstrate high accuracy, with correlation coefficients exceeding *R* ^2^ > 0.95. Ultraviolet absorption is predominantly attributed to HOMO → LUMO transitions, although medycillin exhibits a significant contribution from HOMO‐1 → LUMO excitation, both of which are classified as *π* → π* transitions. Molecular surface ESP analysis reveals maxima near hydrogen atoms of carboxyl and hydroxyl groups and minima around oxygen atoms of ester and carbonyl functionalities. FMO analysis identifies compound **3** as the most reactive species. Compounds **6** and **9** exhibit enhanced chemical stability, as evidenced by lower chemical potential, higher electronegativity, and higher electrophilicity index and hardness. PK evaluations suggest limited intestinal absorption for compounds **8** and **9** due to poor Caco‐2 permeability, high PPB for compounds **2** and **3**, and superior metabolic stability for compound **7** in human liver microsomes. Overall, compound **1** exhibits the highest structural and physicochemical similarity to midecamycin, and molecular docking and MD results confirm its distinct binding affinity, despite a slightly higher binding energy.

## Author Contributions

**Dilong Li:** data curation (equal); formal analysis (equal); investigation (equal); software (equal); writing – original draft (lead). **Yanni Wang:** formal analysis (equal); funding acquisition (lead); investigation (equal); resources (equal). **Yinhuan Huang:** investigation (lead); software (equal). **Hui Zhou:** investigation (equal); software (equal). **Xiaoyun Xia:** investigation (supporting); software (equal). **Wei Huang:** data curation (equal); formal analysis (equal); funding acquisition (equal); validation (equal). **Chaojie Wang:** conceptualization (lead); data curation (lead); formal analysis (lead); methodology (lead); resources (lead); software (lead); validation (lead); writing – review & editing (lead).

## Supporting Information

Additional supporting information can be found online in the Supporting Information Section. **Supporting** **Table** **S1:** Optimized bond lengths (Å) and bond angles (°) of the compound **1** in vacuum using the ωB97XD/6‐311+G(2d, p) method, along with experimental values of bond lengths (Å) and bond angles (°). **Supporting** **Table** **S2:** Theoretical (ωB97XD/6‐311+G(2d, p) calculation in vacuum) and experimental IR vibrational frequencies (in cm^‐1^) of ten 16‐membered lactone ring compounds . **Supporting Table S3:** Theoretial and experimental ^13^C and ^1^H NMR chemical shift data of 16‐membered lactone ring compounds with potential antibacterial activity derived from extreme microbial secondary metabolites in methanol(1‐9) and chloroform(1) solution.

## Funding

This work was supported by the Medical Health Science Technology (2024KY1633); Wenzhou Science and Technology Bureau (Y2023933, Y20220913); Department of Education of Zhejiang Province (Y201942340).

## Conflicts of Interest

The authors declare no conflicts of interest.

## Supporting information

Supplementary Material

## Data Availability

Data are available from the corresponding author upon justified request.
