# Supplementary material for: Computational Study on Potentially Active Antibacterial Compounds in Secondary Metabolites of Extremophilic Microorganisms
Source: ChemistryOpen. 2025 Nov 28;15(4):e202500460. doi: 10.1002/open.202500460 (PMC13052262; doi:10.1002/open.202500460)
Supplement: Supplementary file 1 — Supplementary Material [file OPEN-15-e202500460-s001.pdf]

Supporting Information

Computational Study on Potentially Active Antibacterial Compounds in Secondary Metabolites of Extremophilic Microorganisms

Dilong Li[#], Yanni Wang[#], Yinhuan Huang, Hui Zhou, Xiaoyun Xia, Wei Huang[*], Chaojie Wang [*]

D. Li, H. Zhou, H. Huang
Pharmacy Department
Ruian Hospital of Traditional Chinese Medicine
Wenzhou 325200, Zhejiang, China
E-mail: huangwei1862022@163.com

Y. Wang
Pharmacy Department,
The Third Affiliated Hospital of Wenzhou Medical University
Wenzhou 325200, Zhejiang, China

Y. Huang
Department of Public Health,
Ruian Hospital of Traditional Chinese Medicine
Wenzhou 325200, Zhejiang, China

X. Xia, C. Wang
School of Pharmaceutical Sciences
Wenzhou Medical University
Wenzhou 325035, Zhejiang, China
E-mail: chjwang@wmu.edu.cn

[#] Dilong Li and Yanni Wang contributed equally to this work
*Corresponding authors.

Contents:

Table S1 Optimized bond lengths (Å) and bond angles (°) of the compound **1** in vacuum using the ωB97XD/6-311+G(2d, p) method, along with experimental values of bond lengths (Å) and bond angles (°) .[4]
Table S2 Theoretical (ωB97XD/6-311+G(2d, p) calculation in vacuum) and experimental IR vibrational frequencies (in cm^{-1}) of ten 16-membered lactone ring compounds.[4,51]
Table S3 Theoretial and experimental ^{13}C and ^{1}H NMR chemical shift data of 16-membered lactone ring compounds with potential antibacterial activity derived from extreme microbial secondary metabolites in methanol(**1-9**) and chloroform(**1**) solution.[4]

Table S1 Optimized bond lengths (Å) and bond angles (°) of the compound **1** in vacuum using the ωB97XD/6-311+G(2d, p) method, along with experimental values of bond lengths (Å) and bond angles (°) .[4]

**Bond length**	**Theo./Å**	**Exp./Å**	**Difference**	**Bond angles**	**Theo./°**	**Exp./°**	**Difference**
C8-O24	1.448	1.468	-0.020	C7-C9-O15	111.2	108.1	3.1
C9-O15	1.415	1.426	-0.011	C9-C10-O16	117.9	120.3	-2.4
C10-O16	1.211	1.215	-0.004	C11-C12-S17	113.9	110.1	3.8
C12-S17	1.835	1.830	0.005	C12-C13-O14	124.3	125.3	-1.0
C13-O14	1.204	1.208	-0.004	C12-S17-C18	102.6	101.0	1.6
C13-O24	1.328	1.342	-0.014	C18-C19-O23	112.2	111.3	0.9
O15-H47	0.966	0.755	0.211	C19-C20-O21	123.9	124.6	-0.7
S17-C18	1.829	1.812	0.017	C19-C20-O22	112.5	110.0	2.5
C19-O23	1.395	1.423	-0.028				
C20-O21	1.203	1.205	-0.002				
C20-O22	1.333	1.332	0.001				
O22-H51	0.966	0.854	0.112				
O23-H52	0.970	0.764	0.206				

Table S2 Theoretical (ωB97XD/6-311+G(2d, p) calculation in vacuum) and experimental IR vibrational frequencies (in cm^{-1}) of ten 16-membered lactone ring compounds.[4,51]

Compd.	Exp./cm^{-1}	Theo./cm^{-1}	IR Inten./km/mol	Assignments
**1**	3443	3549	248	-OH (ν_s)
	2932	2931	26	-CH (ν_s, ν_{as})
	2860	2886	5	-CH (ν_s)
	1716	1719	97	-C=O(ν_s)
	1277	1279	3	-CH (τ , ω)
	1234	1234	17	-CH (τ)
	1170	1169	96	-CH (τ)
	1094	1084	16	-CH (ρ)
**2**	3436	3367	828	-OH (ν_s)
	3028	3012	4	-CH (ν_s)
	2933	2933	12	-CH (ν_{as})
	2860	2886	15	-CH (ν_s)
	1726	1725	214	-C=O(ν_s)
	1459	1454	8	-CH (δ)
	1375	1376	2	-CH (ω)
	1268	1265	127	-CH (τ)
	1167	1168	13	-CH (τ)
	1091	1090	10	-CH (ρ)
	909	911	22	-CH (ρ)
**3**	3440	3443	460	-OH (ν_s)
	3020	3015	6	-CH (ν_{as})
	2835	2879	11	-CH (ν_s)
	1745	1744	162	-C=O (ν_s)
	1715	1733	350	-C=O (ν_s)
	1287	1284	9	-CH (τ)
	1048	1049	14	-CH (ω)
**4**	3416	3469	415	-OH (ν_s)
	2928	2929	21	-CH (ν_s)
	1744	1747	449	-C=O (ν_s)
	1702	1733	308	-C=O (ν_s)
	1288	1282	4	-CH (ω)
	1163	1161	25	-CH (τ)
	1043	1044	27	-CH (τ)
**5**	3274	3469	416	-OH (ν_s)
	2914	2912	23	-CH (ν_s)
	1739, 1739	1741, 1733	160, 325	-C=O (ν_s)
	1366	1365	7	-CH (ω)
	1217	1218	132	-CH (τ), -OH (ρ)
**6**	3444	3660	90	-OH (ν_s)
	3020	3026	2	-CH (δ)
	1737	1757	284	-C=O (ν_s)
	1727	1726	246	-C=O (ν_s)

	1366	1368	105	-CH (ω)
	1047	1046	11	-CH (ω)
**7**	3200	3086	1	-CH (ν_s)
	2916	2918	8	-CH (ν_s)
	2850	2875	54	-CH (ν_s)
	1706	1729	240	-C=O (ν_s)
	1275	1278	2	-CH (τ)
	1043	1041	22	-CH (ω)
	732	730	12	-CH (ρ)
**8**	3421	3602	161	-OH (ν_s)
	3020	3019	5	-CH (ν_{as})
	1717	1718	281	-C=O (ν_s)
	1423	1425	2	-CH (δ)
	1170	1174	3	-CH (ρ)
	1044	1048	111	-CH (ω)
	929	928	8	-CH (τ)
**9**	3403	3661	93	-OH (ν_s)
	3020	3031	1	-CH (ν_s)
	1716	1722	237	-C=O (ν_s)
	1508	1444	6	-CH (δ)
	1423	1426	7	-CH (δ)
	1047	1042	7	-CH (ω)
	929	923	1	-CH (ρ)
**M**	3500	3589	146	-OH (ν_s)
	3000	3000	23	-CH (ν_s)
	1760	1759	392	-C=O (ν_s)
	1470	1469	8	-CH (δ)
	1400	1403	14	-CH (δ)
	1190	1190	47	-CH (τ)
	1020	1019	58	-CH (ρ)
	890	892	6	-CH (ρ)

* ν_s is symmetric stretching vibration; ν_{as} is asymmetric stretching vibration; ρ is planar rocking vibration; δ is planar rocking vibration; τ is twisting vibration; ω is out-of-plane rocking vibration

Table S3 Theoretial and experimental ^{13}C and ^{1}H NMR chemical shift data of 16-membered lactone ring compounds with potential antibacterial activity derived from extreme microbial secondary metabolites in methanol(**1-9**) and chloroform(**1**) solution.[4]

**1**	**Theo.**	**Exp.**	**2**	**Theo.**	**Exp.**	**3**	**Theo.**	**Exp.**	**4**	**Theo.**	**Exp.**
	CH_3OH			CH_3OH			CH_3OH			CH_3OH	
C1	28.5	27.2	C1	31.8	27.2	C1	82.0	79.5	C1	79.0	79.3
C2	31.4	26.4	C2	29.3	26.6	C2	206.6	197.7	C2	205.7	197.5
C3	30.9	28.1	C3	31.3	28.3	C3	141.4	137.3	C3	142.2	137.3
C4	28.9	28.1	C4	31.3	27.8	C4	141.4	133.3	C4	141.8	133.0
C5	34.6	27.9	C5	30.5	27.9	C5	29.0	30.1	C5	30.5	30.0
C6	28.1	22.9	C6	28.7	23.4	C6	30.9	28.4	C6	29.7	28.1
C7	37.7	33.6	C7	31.8	30.6	C7	34.2	28.3	C7	34.0	28.1
C8	77.4	73.4	C8	79.5	73.1	C8	22.9	23.5	C8	23.3	24.9
C9	80.2	76.8	C9	83.1	78.7	C9	31.0	28.7	C9	28.9	23.7
C10	227.2	210.2	C10	215.6	205.8	C10	31.2	29.0	C10	30.4	29.3
C11	47.9	43.1	C11	49.2	43.6	C11	33.9	29.3	C11	34.0	28.5
C12	47.0	42.4	C12	50.4	41.8	C13	82.6	74.0	C13	79.8	76.0
C13	181.5	174.2	C13	180.5	174.2	C14	32.8	35.6	C14	73.8	74.8
C18	45.0	36.7	C18	43.3	36.7	C15	32.5	24.8	C15	43.5	33.4
C19	76.8	71.5	C19	76.1	71.5	C16	171.5	166.5	C16	171.3	166.2
C20	182.4	175.9	C20	180.7	176.0	C18	181.7	173.6	C18	180.1	173.6
C25	34.0	36.1	C25	30.3	36.0	C19	36.1	29.8	C19	35.6	29.8
C26	21.5	24.5	C26	23.5	24.4	C20	35.0	29.9	C20	33.0	29.9
C27	18.4	20.4	C27	19.9	20.3	C21	182.1	175.9	C21	182.5	175.8
H28	0.9	1.3	C28	185.2	176.1	C25	22.9	20.2	C26	20.8	20.6
H29	1.5	1.3	C30	32.1	29.9	H28	4.7	5.4	H29	5.5	5.3
H30	1.1	1.3	C31	30.1	29.8	H29	7.9	7.3	H30	8.1	7.3
H31	1.2	1.3	C32	180.9	173.7	H30	7.1	6.7	H31	7.2	6.7
H32	1.2	1.3	H35	1.5	1.3	H31	2.0	2.0	H32	2.2	1.9
H33	1.2	1.3	H36	0.7	1.3	H32	1.3	1.9	H33	1.2	1.5
H34	0.8	1.3	H37	1.1	1.3	H33	1.6	1.3	H34	1.3	1.3
H35	1.6	1.3	H38	1.1	1.3	H34	1.4	1.3	H35	1.2	1.3
H36	1.8	1.3	H39	1.4	1.3	H35	1.1	1.3	H36	0.9	1.3
H37	0.8	1.3	H40	1.3	1.3	H36	1.6	1.3	H37	1.4	1.3
H38	1.1	1.1	H41	1.1	1.3	H37	1.6	1.3	H38	0.9	1.5
H39	1.9	1.3	H42	1.4	1.3	H38	1.7	1.3	H39	1.7	1.5
H40	0.6	1.8	H43	1.5	1.3	H39	1.1	1.3	H40	1.0	1.3
H41	2.0	1.8	H44	0.9	1.3	H40	1.9	1.3	H41	1.7	1.3
H42	4.9	5.0	H45	1.4	1.3	H41	1.2	1.3	H42	1.2	1.3
H43	4.2	4.3	H46	2.1	1.3	H42	1.7	1.3	H43	2.2	1.3
H44	3.9	3.2	H47	1.8	1.9	H43	1.7	1.3	H44	1.7	1.3
H45	2.8	2.9	H48	1.6	1.9	H44	0.9	1.3	H45	0.9	1.3
H46	4.0	3.9	H49	4.7	5.0	H45	5.3	5.1	H46	5.5	4.9
H47	2.7		H50	5.2	5.2	H46	1.5	1.6	H47	4.3	3.5
H48	3.5	3.2	H51	3.7	3.2	H47	2.6	1.7	H48	1.4	1.4

H49	2.9	2.9	H52	3.2	2.9	H48	1.6	1.3	H49	2.0	1.6
H50	4.9	4.4	H53	3.9	3.9	H49	1.6	1.3	H50	2.5	2.7
H51	6.5		H54	3.5	3.2	H50	2.7	2.7	H51	3.2	2.7
H52	5.5		H55	3.3	2.9	H51	2.9	2.7	H52	2.5	2.6
H53	1.9	1.6	H56	3.8	4.4	H52	2.8	2.6	H53	2.6	2.6
H54	1.6	1.4	H57	9.1		H53	2.3	2.6	H54	8.5	
H55	1.4	1.3	H58	1.8		H54	8.6		H55	0.6	
H56	1.2	1.3	H59	2.4	1.6	H55	1.2	1.3	H56	1.8	1.4
H57	1.0	1.3	H60	1.3	1.4	H56	1.4	1.3	H57	1.2	1.4
H58	1.3	1.3	H61	1.8	1.3	H57	1.3	1.3	H58	1.4	1.4
H59	1.4	1.3	H62	1.1	1.3						
			H63	0.9	1.3						
			H64	1.4	1.3						
			H65	1.7	1.3						
			H66	2.9	2.6						
			H67	2.9	2.6						
			H68	2.7	2.7						
			H69	2.8	2.7						
			H70	6.3							

**5**	**Theo.**	**Exp.**	**6**	**Theo.**	**Exp.**	**7**	**Theo.**	**Exp.**	**8**	**Theo.**	**Exp.**
	CH_3OH			CH_3OH			CH_3OH			CH_3OH	
C1	79.4	79.3	C1	82.4	77.8	C1	75.8	75.2	C1	84.8	77.9
C2	205.2	197.9	C2	74.2	73.0	C2	74.6	75.7	C2	78.4	74.6
C3	142.9	137.9	C3	151.5	148.3	C3	154.2	149.9	C3	153.8	148.7
C4	141.4	133.2	C4	132.4	123.3	C4	130.5	122.5	C4	129.9	123.1
C5	30.1	30.0	C5	30.9	30.4	C5	32.6	30.3	C5	32.5	30.4
C6	29.5	28.5	C6	31.8	28.6	C6	31.3	27.5	C6	30.8	29.1
C7	33.9	28.5	C7	31.2	27.3	C7	31.6	28.9	C7	34.5	27.4
C8	23.1	23.4	C8	25.1	24.8	C8	23.7	25.0	C8	23.8	24.1
C9	36.5	36.8	C9	28.4	27.6	C9	27.8	24.1	C9	29.8	24.9
10	21.0	26.2	C10	29.9	28.4	C10	30.4	27.3	C10	31.0	27.5
C11	33.4	29.2	C11	32.0	27.5	C11	32.2	29.1	C11	33.9	27.6
C13	77.2	71.0	C13	78.4	72.5	C13	79.0	74.6	C13	82.5	75.1
C14	43.9	42.6	C14	33.1	36.8	C14	73.0	75.0	C14	73.5	72.9
C15	75.4	68.1	C15	32.0	25.3	C15	43.6	33.3	C15	41.7	33.4
C16	171.5	166.2	C16	173.8	167.8	C16	173.5	167.5	C16	174.5	167.3
C18	180.1	173.6	C17	22.8	20.9	C18	19.7	18.2	C18	18.5	18.2
C19	35.6	29.8	C20	181.6	174.2	H22	4.1	3.6	C21	184.1	174.2
C20	32.6	29.9	C22	32.7	29.3	H23	4.1	4.4	C23	35.0	28.7
C21	182.4	175.9	C23	33.3	29.9	H24	7.4	7.0	C24	37.5	29.9
C26	23.0	20.6	C24	180.5	176.2	H25	6.7	6.1	C25	180.5	176.2
H29	5.5	5.4	H28	5.0	4.8	H26	1.4	1.3	H29	4.7	4.8
H30	8.1	7.3	H29	4.2	4.6	H27	1.7	1.6	H30	4.2	4.6

H31	7.2	6.7	H30	7.3	6.9	H28	1.7	1.3	H31	7.3	7.0
H32	2.1	2.0	H31	6.6	6.1	H29	0.9	1.3	H32	6.5	6.1
H33	1.3	1.8	H32	1.7	1.6	H30	0.9	1.3	H33	2.1	1.7
H34	1.5	1.3	H33	1.9	1.6	H31	1.6	1.3	H34	1.5	1.5
H35	1.3	1.3	H34	1.8	1.3	H32	1.4	1.3	H35	1.7	1.3
H36	1.0	1.3	H35	0.8	1.3	H33	1.6	1.3	H36	1.2	1.3
H37	1.4	1.3	H36	1.0	1.5	H34	1.1	1.3	H37	1.0	1.3
H38	0.9	1.2	H37	1.5	1.7	H35	1.8	1.3	H38	1.7	1.3
H39	1.7	1.5	H38	1.0	1.3	H36	1.4	1.3	H39	1.3	1.3
H40	1.4	1.3	H39	1.8	1.3	H37	1.4	1.3	H40	1.7	1.3
H41	1.9	1.3	H40	1.1	1.3	H38	1.7	1.3	H41	1.2	1.3
H42	1.7	1.3	H41	1.7	1.3	H39	0.8	1.3	H42	2.3	1.3
H43	2.0	1.3	H42	1.2	1.3	H40	4.9	4.8	H43	1.3	1.3
H44	1.7	1.3	H43	1.4	1.3	H41	3.9	3.4	H44	1.4	1.3
H45	0.9	1.3	H44	1.6	1.3	H42	1.5	1.4	H45	1.8	1.3
H46	5.5	5.2	H45	0.9	1.3	H43	1.9	1.6	H46	0.9	1.3
H47	1.4	1.8	H46	5.0	5.1	H44	0.3		H47	4.4	4.8
H48	2.9	1.9	H47	1.4	1.3	H45	1.6	1.3	H48	3.7	3.4
H49	4.1	3.8	H48	1.9	1.3	H46	1.0	1.3	H49	1.6	1.3
H50	2.5	2.7	H49	1.5	1.3	H47	1.2	1.3	H50	1.9	1.4
H51	3.3	2.7	H50	1.6	1.3	H48	1.2		H51	0.3	
H52	2.5	2.6	H51	1.1	1.3	H49	2.1		H52	1.6	1.3
H53	2.6	2.6	H52	1.1	1.3				H53	1.7	1.3
H54	8.4		H53	1.4	1.3				H54	1.6	1.3
H55	0.8		H54	2.8	2.7				H55	2.3	2.6
H56	1.3	1.4	H55	3.0	2.7				H56	3.1	2.6
H57	1.4	1.4	H56	2.8	2.7				H57	3.1	2.6
H58	1.3	1.4	H57	2.5	2.7				H58	2.7	2.6
			H58	6.4					H59	6.5	
			H59	1.3					H60	3.8	

**9**	**Theo.**	**Exp.**	**1**	**Theo.**	**Exp.**
	CH_3OH			$CHCl_3$	
C1	82.4	77.8	C1	28.7	25.3
C2	74.3	73.0	C2	31.2	26.6
C3	152.4	148.7	C3	31.4	26.6
C4	131.8	123.1	C4	29.1	26.6
C5	30.9	29.3	C5	33.7	26.0
C6	31.7	27.6	C6	28.1	20.7
C7	31.2	27.3	C7	41.8	32.3
C8	25.0	25.2	C8	75.0	73.3
C9	28.9	28.6	C9	78.8	76.2
C10	29.9	28.6	C10	223.3	208.8
C11	32.0	27.6	C11	51.2	40.9

C13	80.0	76.5	C12	48.3	41.3
C14	28.8	31.5	C13	179.6	172.3
C15	31.7	25.0	C18	33.3	35.7
C16	173.7	168.1	C19	73.8	70.4
C17	66.4	65.1	C20	180.3	174.9
C21	181.7	174.2	C25	36.6	34.5
C23	31.6	30.0	C26	22.6	22.9
C24	30.8	30.5	C27	18.0	19.8
C25	181.8	176.3	H28	0.9	1.3
H29	5.0	4.8	H29	1.6	1.3
H30	4.2	4.6	H30	1.4	1.3
H31	7.4	7.0	H31	1.3	1.3
H32	6.6	6.2	H32	1.2	1.3
H33	1.7	1.5	H33	1.3	1.3
H34	1.8	1.7	H34	0.9	1.3
H35	1.7	1.3	H35	1.7	1.3
H36	0.8	1.3	H36	1.9	1.3
H37	0.9	1.3	H37	0.9	1.3
H38	1.5	1.3	H38	1.1	1.0
H39	1.0	1.3	H39	1.9	1.4
H40	1.7	1.3	H40	0.9	1.8
H41	1.1	1.3	H41	2.2	1.8
H42	1.7	1.3	H42	5.2	4.9
H43	1.3	1.3	H43	4.4	4.4
H44	1.4	1.3	H44	3.8	3.2
H45	1.6	1.3	H45	3.1	2.8
H46	0.9	1.3	H46	4.6	4.0
H47	4.9	5.1	H47	3.2	
H48	2.0	1.6	H48	3.5	3.3
H49	1.7	1.5	H49	2.9	3.0
H50	1.6	1.3	H50	4.2	4.5
H51	1.6	1.3	H51	6.4	
H52	3.8	3.6	H52	1.7	
H53	3.5	3.6	H53	1.3	1.6
H54	0.7		H54	1.8	1.4
H55	2.8	2.6	H55	1.6	1.3
H56	2.6	2.6	H56	1.3	1.3
H57	2.7	2.6	H57	0.9	1.3
H58	2.8	2.6	H58	1.3	1.3
H59	6.3		H59	1.3	1.3
H60	1.3				

References:

[4] Stierle, D. B. Stierle, D. Decato, N. D. Priestle, J. B. Alverson, J. Hoody, K. McGrath, K. Klepacki, *J. Nat. Prod.* **2017**, *80*, 1150.

[51] Tsuruoka, T.; Shomura, T.; Ezaki, Watanabe H.; Akita, E. *J. Antibiot(Tokyo)*. **1971**, *24*(7): 452-459.
